# *S*-palmitoylation of MTDH regulates ferroptosis resistance in breast cancer cell

**DOI:** 10.1016/j.jlr.2025.100953

**Published:** 2025-11-28

**Authors:** Shaojun Pei, Wen Wang, Tingze Feng, Qiuping Wang, Yuhan Wang, Hong-Xu Liu, Xinmiao Liang, Hai-long Piao

**Affiliations:** 1 State Key Laboratory of Phytochemistry and Natural Medicines, Dalian Institute of Chemical Physics, Chinese Academy of Sciences, Dalian, China; 2 University of Chinese Academy of Sciences, Beijing, China; 3 Department of Neurology, The First Affiliated Hospital of Anhui Medical University, Hefei, Anhui, China; 4 Department of Thoracic Surgery, Cancer Hospital of Dalian University of Technology, Liaoning Cancer Hospital & Institute, Shenyang, China; 5 Department of Biochemistry & Molecular Biology, School of Life Sciences, China Medical University, Shenyang, China

**Keywords:** *S*-palmitoylation, MTDH, lipidomics, ferroptosis, breast cancer

## Abstract

*S*-palmitoylation is a dynamic and reversible post-translational modification that plays crucial roles in cancer progression. Here, we found that the oncogene of metadherin (MTDH) modulates lipid metabolism and ferroptosis by its *S*-palmitoylation. We demonstrate that MTDH is *S*-palmitoylated at Cys-75 in the endoplasmic reticulum by ZDHHC1/9 and *S*-depalmitoylated by APT1. The flexible loop and the α-helix length in the MTDH N-terminus affect its *S*-palmitoylation level. In addition, metabolomics analysis found that the *S*-palmitoylated MTDH increases intracellular levels of triglycerides, phosphatidylethanolamines, and phosphatidylcholines. The non-*S*-palmitoylation form of MTDH-CS enhanced the interaction between MTDH and the ferroptosis enhancer of Acyl-CoA synthetase long-chain family member 4 (ACSL4), thereby reducing ferroptosis sensitivity in breast cancer cells.

*S*-palmitoylation is a reversible post-translational modification (PTM) in eukaryotes, introducing long chain fatty acids into cysteine residues of proteins. *S*-palmitoylation is catalyzed by palmitoyl *S*-acyltransferases (PATs, also known as ZDHHC proteins) by the ping-pong mechanism (1). In mammalian cells, 23 members of PATs share a similar topology, which is characterized by an Asp-His-His-Cys (DHHC) motif within the cysteine-rich domain and at least four transmembrane domains (TMDs) (1). Removing the fatty acyl chains from the substrates, *S*-depalmitoylation is catalyzed by the protein thioesterases (APTs). Proteins undergo *S*-palmitoylation increasing the affinity to the membrane, which influence proteins conformation, organization, localization, and stability (2). Various physiological and pathological processes, such as metabolism, innate immunity, cell death, nervous system diseases and cancer are intimately related to *S*-palmitoylation of substrates (3).

Lipids could noncovalently interact with proteins and covalently modify proteins through PTMs, affecting protein function (4). Our previous research has established that metadherin (MTDH) could interact with cholesterol (5). Meanwhile, MTDH was found in *S*-palmitoylation proteomic studies (6, 7). *S*-palmitoylation of MTDH is verified in hepatocellular carcinoma (HCC) cells by the acyl resin-assisted capture method (8). MTDH is a single transmembrane protein, which has been observed to be located in the plasma membrane, nucleus, and endoplasmic reticulum (ER) (9). MTDH is an oncogene highly expressed in various cancers, such as breast invasive carcinoma (BRCA), non-small cell lung cancer (NSCLC), and HCC (9, 10). MTDH is associated with tumor metastasis, drug resistance, and lipid metabolism (10, 11). MTDH *S*-palmitoylation regulates its protein stability and weakens the interaction between MTDH and staphylococcal nuclease domain-containing protein 1 (SND1) (8). Restraining MTDH *S*-palmitoylation promotes the progression of DEN-induced HCC in vivo (8). However, it is still unknown whether MTDH *S*-palmitoylation regulates metabolic homeostasis in cancer cells.

Programmed cell death (PCD) is genetically regulated, and it is categorized as apoptosis, autophagy, pyroptsis, necroptosis, and ferroptosis (12). Ferroptosis is an iron-dependent form of cell death caused by accumulation of peroxidation lipid (13). Acyl-CoA synthetase long-chain family member 4 (ACSL4) facilitates the incorporation of polyunsaturated fatty acids (PUFAs) into phospholipids (PLs), enhancing the peroxidation susceptibility of membranes (14). Glutathione peroxidase 4 (GPX4) utilizes glutathione (GSH) to reduce lipid hydroperoxides, preventing peroxidation lipid accumulation (13). GPX4 is the only enzyme to reduce toxic lipids, playing a vital role in preventing ferroptosis. The *S*-palmitoylation of GPX4 facilitates its stability and enhances ferroptosis resistance in tumor cells (15, 16). Solute carrier family 7 member 11 (SLC7A11) transports extracellular cystine into the cells, maintaining intracellular GSH level to regulate GPX4 activity (13). The *S*-palmitoylation of SLC7A11 increases its stability and strengthens tumor cells' resistance to ferroptosis (17, 18). Together, *S*-palmitoylation of ferroptotic factors will enhance the ability of ferroptosis resistance of cells. In addition, *S*-palmitoylation of the ubiquitin-specific proteinase (USP) family indirectly regulates the degradation of GPX4 and ACSL4 to influence the ferroptosis resistance of cancer cells (19, 20). However, the role of *S*-palmitoylation in ferroptosis still remains unclear, and its mechanisms require further investigation.

In this study, we demonstrate that MTDH is reversibly *S*-palmitoylated at cysteine 75 in ER, catalyzed by ZDHHC1/9, and APT1. The N-terminal of MTDH influences its *S*-palmitoylation catalytic process. Moreover, we uncovered that the *S*-depalmitoylation form of MTDH increased the interaction with ACSL4, thereby alters the ACSL4 activity in ferroptosis process. These findings reveal a novel mechanism axis involving MTDH *S*-palmitoylation and ACSL4 in ferroptosis.

## Materials and methods

### Cell culture

HEK293T, HeLa, MCF7, MDA-MB-231, MDA-MB-453, MDA-MB-361, and BT549 cells were cultured in DMEM (Gibco). HCC1937 and SUM159PT cells were cultured in RPMI 1640 (Gibco). Cell lines were maintained in culture supplemented with 10% fetal bovine serum (FBS, Gibco) and 1% penicillin/streptomycin (Selleck, E2894) at 37°C with 5% CO_2_ in a humidified incubator (ThermoFisher Scientific).

### Plasmid cloning

For transient transfection, full-length, mutated and truncated MTDH, ZDHHC family, APT1, APT2, ABHD17A/B/C, ABHD10, PPT1, and ACSL4 coding sequences were fused with SF and HA tag sequences by PCR and then subcloned to the pCDNA3.1 vector in appropriate restriction endonuclease sites. The targeted sequences were shown in the Supplemental Table.

For the stable expression of proteins and shRNAs in cells, the lentivirus packaging system was used. Coding sequences of MTDH and MTDH-CS mutant were subcloned to pLoc vector in appropriate restriction endonuclease sites. Lentiviral shRNAs were cloned in pLKO.1 vector within the AgeI/EcoRI sites at the 3′ end of the human U6 promoter. The targeted sequences were shown in the Supplementary Table.

### Transfection and lentivirus infection

HEK293T cells were used for transfection and lentivirus packaging. Plasmids and PEI (polyethylenimine, Polysciences, 24,765) were mixed at 1:4 ratio (w/w) in Opti-MEM medium (Gibco) and then added to HEK293T cell medium. Protein expression was tested about 48 h after transfection.

For lentivirus packaging, the protein or shRNA expressing lentivirus vectors were transfected into HEK293T cells together with packaging vectors psPAX2 and pVSVG using PEI. Virus-containing medium was harvested 48 h after transfection and filtered with 0.22 μm membrane (Millipore, SLGP033RB) to remove cell debris, then the virus-containing medium was used to culture target cells for 24 h. Puromycin (3 μg/ml, InvivoGen, ant-pr-1) or Blasticidin (8 μg/ml, InvivoGen, ant-bl-5) were used to screen positive cells, depending on the lentivirus vector resistant gene.

### 17-ODYA labeling and click reaction

Metabolic labeling of bioorthogonal chemical reporters of protein and detection of protein *S*-palmitoylation via click chemistry were performed as previously described with minor modifications (6, 7). For labeling, the cells were incubated with FBS free DMEM containing 100 μM palmitic acid (blank control) or 17-ODYA (MedChemExpress, HY-101016) for 12 h. After labeling overnight, cells were washed with cold PBS and resuspended in Lysis Buffer (50 mM Tris-HCl, pH 7.4, 150 mM NaCl, 4 mM EDTA, 0.5% NP-40) supplemented with 0.6% SDS, protease inhibitors cocktail (Selleck, B14002) and phosphatase inhibitors cocktail (Selleck, B15002). The lysates were centrifuged at 14,000 × g for 15 min at 4°C, and the protein concentrations of the supernatants were measured using BCA protein assay kit II (Abcam, ab287853). Taking 1 mg total proteins and incubating with 1 mM CuSO4, 1.5 mM TCEP, 100 μM TBTA, and 200 μM biotin-azide (Aladdin, B152153) at room temperature for 2 h. Then, the proteins were precipitated by methanol-chloroform-water (MCW) precipitation and dried the pellet. The dried protein pellet was dissolved in 100 μl Lysis Buffer supplemented with 4% SDS, protease and phosphatase inhibitors. Then, adding 900 μl Lysis Buffer supplemented with protease and phosphatase inhibitors and incubating with streptavidin beads 6FF (Smart-Lifesciences, SA021005) at 4°C overnight with rotation. Unbound proteins were removed by washing with Lysis Buffer for three times. Bound proteins were eluted by boiling the beads at 98°C for 10 min with 1× SDS-PAGE loading buffer (Takara, 9173). The samples were then analyzed by immunoblotting assay.

### Acyl-PEG exchange (APE) and acyl-biotin exchange (ABE)

The APE and ABE assay was performed as previously described with minor modifications (21, 22). In brief, cells were scraped from plates and lysed in Lysis Buffer supplemented with 0.6% SDS, protease, and phosphatase inhibitors. The lysates were centrifuged at 14,000 × g for 15 min at 4°C, and the protein concentrations of the supernatants were measured using BCA protein assay kit II. Taking 800 μg total proteins and incubating with 10 mM TCEP at room temperature for 30 min. Then, add freshly prepared N-ethylmaleimide (NEM, Sigma-Aldrich, E3876) dissolved in ethanol to achieve a final concentration of 25 mM, and incubate the mixture at room temperature for 2 h. After incubation, the proteins were precipitated by MCW precipitation and dried the pellet. Then, dissolving the dried protein pellet in 190 μl Lysis Buffer supplemented with 4% SDS and equivalently divided the samples equally into two tubes. For the hydroxylamine (HAM, Macklin, H828371) treatment, adding 5 μl HAM into one group (+HAM) and 5 μl H_2_O into the other (-HAM, control group), and incubating the mixture at room temperature for 1 h. After that, the proteins were precipitated by MCW precipitation and dried in the pellet.

For the APE assay, dissolve the dried protein pellet in 70 μl Lysis Buffer supplemented with 4% SDS. and add 10 μl 8 mM methoxy-PEG-maleimide (average Mn = 10 kD, Bidepharm, BD01161968) to achieve a final concentration of 1 mM. Incubating the mixture at room temperature for 2 h. Then, the samples were mixed with SDS-PAGE loading buffer and denatured at 98°C for 10 min and analyzed by immunoblotting assay. After that, the relative protein abundance was analyzed using ImageJ (version 1.54f) grayscale analysis. The *S*-palmitoylation ratio is calculated by dividing the *S*-palmitoylated protein abundance by the sum of *S*-palmitoylated and *S*-depalmitoylated protein abundance.

For the ABE assay, dissolving the dried protein pellet in 75 μl Lysis Buffer supplemented with 4% SDS and adding 5 μl 16 mM biotin-maleimide (Sigma-Aldrich, B1267) to achieve a final concentration of 1 mM. Incubating the mixture at room temperature for 2 h. After that, the proteins were precipitated by MCW precipitation and dried the pellet. The dried protein pellet was dissolved in 100 μl Lysis Buffer supplemented with 4% SDS, protease and phosphatase inhibitors. Then, adding 900 μl Lysis Buffer supplemented with protease and phosphatase inhibitors and incubating with streptavidin beads 6FF at 4°C overnight with rotation. Unbound proteins were removed by washing with Lysis Buffer for three times. Bound proteins were eluted by boiling the beads at 98°C for 10 min with SDS-PAGE loading buffer. The samples were then analyzed by immunoblotting assay.

### Immunoblotting

Cells were scraped from plates and lysed in Lysis Buffer supplemented with 0.6% SDS, protease and phosphatase inhibitors. The cell lysates were clarified by centrifugation at 14,000 × g for 15 min at 4°C, and the protein concentrations of the supernatants were measured using BCA protein assay kit II. Total protein was mixed with SDS-PAGE loading buffer and denatured at 98 °C for 10 min. Samples were separated by SDS-PAGE and followed by transfer to a PVDF membrane (Millipore, IPVH00010). Then, the PVDF membranes were blocked with 5% (w/v) nonfat dry milk for 2 h at room temperature. Then, the proteins on the PVDF membrane were immunoblotted with indicated primary antibodies overnight at 4°C, followed by washing in 0.1% Tween/PBS. Membranes were incubated with corresponding secondary antibodies (goat anti-mouse, Millipore, AP-124P, and goat anti-rabbit, Millipore, AP-132P) at room temperature for 2 h and then washed three times before signal detection. Indicated primary antibodies listed in Supplemental Table.

### Immunofluorescence

After seeding cells on the 4-chamber slide, and cells were fixed with 4% paraformaldehyde (PFA) and permeabilized with 0.1% Triton X-100 for 10 min. After blocking with 10% goat serum, the permeabilized cells were incubated with primary antibodies overnight at 4°C. Then, the cells were washed in PBS, stained with secondary antibodies (goat anti-mouse Alexa Fluor™ 488, Invitrogen, A11001, and goat anti-rabbit Alexa Fluor™ 555, Invitrogen, A31572) for 2 h at room temperature, followed by counterstaining with DAPI. Images were taken with a scanning confocal microscope.

### ER isolation

According to the manufacturer's recommendations, adherent cells were scraped, and the cell pellet was obtained by centrifugation. Next, resuspend the cell pellet in 500 μl of Buffer A per 10^7^ cells, and transfer it into a glass Dounce homogenizer. Incubate on ice for 10 min. Cells were homogenized with 30–40 strokes using the Dounce homogenizer, and then the homogenate solution was transferred to a new tube and centrifuged at 1,000 × g at 4°C for 5 min. Carefully transfer the supernatant into a new tube, collect the pellet (P1), and dissolve it in loading buffer. Samples in the new tube were centrifuged at 11,000 × g at 4°C for 10 min. Carefully transfer the supernatant into a new tube, collect the pellet (P2), and dissolve it in loading buffer. Samples in the new tube were centrifuged at 44,700 × g at 4°C for 45 min. Carefully remove the supernatant (S3) and resuspend the pellet in 400 μl of cold Buffer B. Mix the sample and centrifuge at 44,700 × g at 4°C for 45 min. Carefully remove the supernatant (S4) and dissolve the pellet in 200 μl Lysis Buffer (P4).

### Nucleus–cytoplasmic fractionation assay

According to the manufacturer's recommendations, adherent cells were scraped, and the cell pellet was obtained by centrifugation. Next, resuspend cell pellet in 100 μl of 1× Pre-Extraction Buffer per 10^6^ cells, and transfer to a micro-centrifuge vial. Incubate on ice for 10 min. Vortex vigorously for 10 s and centrifuge the preparation for 1 min at 10,000 × g. Carefully remove the cytoplasmic extract from the nuclear pellet. Add Lysis Buffer containing to nuclear pellet. Incubate the extract on ice for 15 min with vortex (5 s) every 3 min. The extract can be further sonicated for 3 times per 10 s to increase nuclear protein extraction for tissue extract. Centrifuge the suspension for 10 min at 14,000 × g at 4°C and transfer the supernatant into a new microcentrifuge vial.

### Ni-NTA agarose purification assay

Briefly, 6×His-tagged MTDH proteins were expressed in BL21 (DE3) *Escherichia coli* via transforming pET24a-MTDH-6×His plasmids. Then, the BL21 cells were collected, sonicated, and purified with BeyoGold™ His-tag purification resin (Beyotime, P2210) to obtain purified MTDH-6×His protein.

### RNA isolation and quantitative RT-PCR

RNA of cell samples was isolated with RNAiso Plus reagent (Takara, 9108). The concentrations of RNA were determined with Nanodrop (ThermoFisher Scientific). Reverse transcription PCR was performed using Hifair® AdvanceFast first Strand cDNA Synthesis Kit (Yeasen, 11150ES60) to obtain cDNA as a template for quantitative RT-PCR. The quantitative RT-PCR was performed using Hieff UNICON® Advanced qPCR SYBR Master Mix (Yeasen, 11185ES03) in the CFX-96 instrument (Bio-Rad). The Ct technique is used to calculate fold changes. All reactions were run in triplicate. Results were normalized to *GAPDH* levels. The primer sequences were as follows:

#### ZDHHC1

F 5′-ATGGAGTTCTACATGCGGACCTTC-3′

R 5′-TGGCAAGAAACTGGGAGGGATTG-3′

#### ZDHHC9

F 5′-CCCAGGCAGGAACACCTTTT-3′

R 5′-CCGAGGAATCACTCCAGGG-3′

#### MTDH

F 5′-AAATAGCCAGCCTATCAAGACTC-3′

R 5′-TTCAGACTTGGTCTGTGAAGGAG -3′

#### GAPDH

F 5′-CATCTTCTTTTGCGTCGCCA-3′

R 5′-TTAAAAGCAGCCCTGGTGACC-3′

### Coimmunoprecipitation

Cells were scraped from dishes and lysed with NETN buffer (20 mM Tris-HCl, pH 8.0, 100 mM NaCl, 1 mM EDTA, 0.5% NP-40) containing protease and phosphatase inhibitors. The cell lysates were clarified by centrifugation at 14,000 rpm for 15 min at 4°C. For coimmunoprecipitation, cell lysates were incubated with the anti-Flag affinity gel (Selleck, B23102) or anti-HA magnetic beads (Bimake, B26202) at 4°C overnight. Then, the agarose was washed 4 times with NETN buffer, followed by adding 1× SDS-PAGE loading buffer and being tested by immunoblotting assay.

### Wound healing assay

Wound healing assay was used to test cell migration abilities. Cells were seeded in 6-well plates. After the cells reached approximately 98% confluency, they were scratched with a sterile 10 μl pipette tip and incubated in fresh serum-free media after the cell debris was removed by PBS. The width of the gap was photographed and measured at the indicated time points with Image J.

### BODIPY 493/503 staining and lipid droplet counting

Lipid droplet levels were measured using BODIPY 493/503 staining (Invitrogen, D3922). Cells were seeded on the 4-chamber slide and cells were incubated with 2 μM BODIPY493/503 for 15 min at 37°C. Then, the cells were fixed with 4% PFA for 10 min, followed by counterstaining with DAPI. Images were taken with a scanning confocal microscope. The lipid droplets were counted by CellProfiler (version 4.2.5) (23).

### Untargeted lipidomics

Lipid extraction from cells was performed as previously described with minor modifications (24, 25). In brief, cells collected in a 10 cm dish were rinsed with PBS and instantly frozen in liquid nitrogen. Cells were then lysed with 700 μl of methanol containing internal standards, and then mixed with 700 μl chloroform and vortexed for 20 s. Subsequently, 280 μl water were added and again for 20 s vortex. The hydrophobic layer was collected and freeze-dried. Quality control (QC) sample was also prepared by combining the organic phase and then freeze-dried to evaluate the analytical quality. The lyophilized powder was redissolved in 20 μl organic solvent (chloroform/methanol = 2/1, v/v) by 30 s vortex, then added 60 μl organic solvent (acetonitrile/isopropanol/water = 65/30/5, v/v, containing 5 mM ammonium acetate). After centrifugation, the supernatant can be directed for the 1290–6546 LC/Q-TOF (Agilent Technologies) analysis.

The acquisition condition for lipidomics analysis was referred from previous study (26). Briefly, ACQUITY UPLC® BEH C8 column (100 mm × 2.1 mm, 1.7 μm, Waters, USA) was used for lipid separation and the column temperature was set to 60°C. Acetonitrile/water (60:40, v/v) and isopropanol/acetonitrile (90:10, v/v) both containing 10 mM ammonium acetate was used as mobile phase. The sample injection volume was 5 μl and multisampler temperature was set to 10°C. Gas temperature was 320°C with the sheath gas temperature at 350°C. Both the negative and the positive mode was used, the m/z scan range was 50–1500 Da, and the fragmentor was 175V. The capillary voltage was 4 kV for the positive mode, while 3.5 kV for the negative mode. For auto-MS/MS, both 15 eV and 30 eV were used as collision energy. MassHunter Acquisition (version 10.1) was used for raw data collection of lipid profiling.

MS-DIAL (version 4.92) was used to compare and process data (27). The differential lipids were identified based on their retention time, accurate mass, and spectrometric fragments. After manually integrating the peak area and deriving Excel table, the data were normalized by the total peak area to obtain the relative concentration.

### RSL3 sensitivity testing

Cell viability was measured using a Cell Counting Kit-8 (CCK-8, Yeasen, 40203ES60). Briefly, cells were seeded in a 96-well plate at a density of 3000 cells per well. The next day, after the treatment of different concentrations of RSL3 (MedChemExpress, HY-100218A) or ML348 for a certain time, each well was added with 10 μl of CCK-8 and cultured for 2 h (37°C, 5% CO_2_), and the absorbance was detected by Cytation5 (BioTek) at 450 nm.

### PI staining

Cells were seeded in a 24-well plate. On the following day, the cells were treated with RSL3 for 24 h, stained with 5 μg/ml propidium iodide (PI, Innochem, B04206) and imaged by a Cytation5.

### Statistical analysis

Data representative of two or more independent experiments. The comparison of indicated two groups was performed by Student's *t* test (two-tailed, unpaired): ∗*P* < 0.05, ∗∗*P* < 0.01, ∗∗∗*P* < 0.001, ∗∗∗∗*P* < 0.001, and not significant (NS). All of the statistical details can be found in the figure legends. Bars and error represent mean ± standard deviations (SD) of replicate measurements. All of the relative protein expression was normalized by ImageJ.

## Result

### MTDH is an *S*-palmitoylated ER-localized protein

**Fig. 1:**
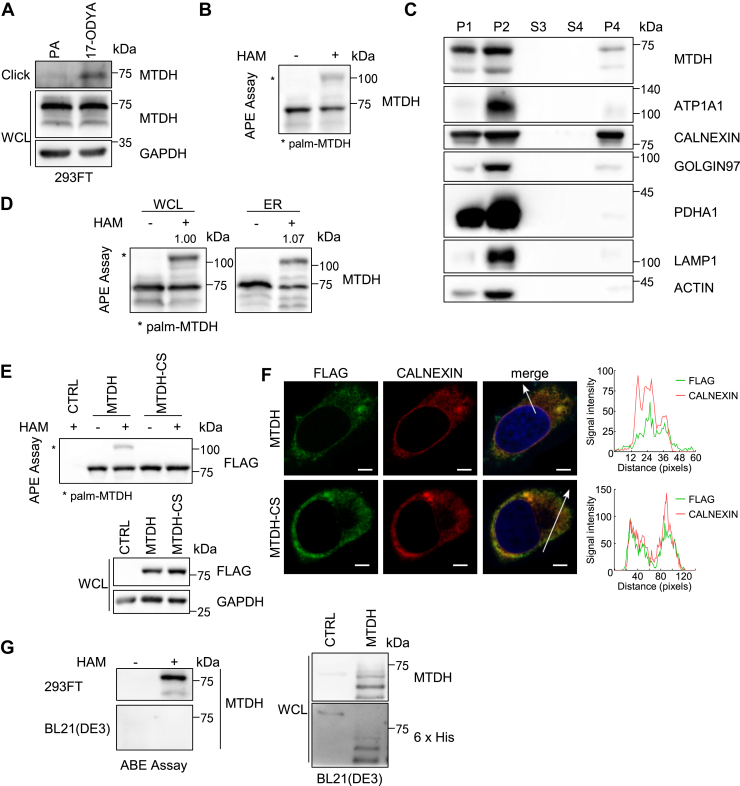
MTDH is an *S*-palmitoylated ER-localized protein. A: Endogenous MTDH *S*-palmitoylation in HEK293T cells was detected using a bioorthogonal labeling approach. B: APE assay detecting endogenous MTDH *S*-palmitoylation in HEK293T cells with (+HAM) or without (−HAM) hydroxylamine treatment. C: ER fractions were purified from HEK293T cells; P4 represents the ER-enriched fraction. D: Comparison of MTDH *S*-palmitoylation levels between whole cell lysates (WCL) and ER-enriched fractions using the APE assay. Data normalized to the WCL group. E: MTDH-WT or palmitoylation-deficient MTDH-CS was expressed in HEK293T cells and analyzed by APE assay. F: Immunofluorescence of MTDH-WT or MTDH-CS (FLAG) and ER marker calnexin in HeLa cells. Scale bar, 5 μm. G: ABE assay detecting *S*-palmitoylation of His-tagged MTDH expressed in E. coli BL21 (DE3), with or without HAM treatment. All experiments were repeated independently at least three times.

To investigate whether MTDH undergoes *S*-palmitoylation, we used the bioorthogonal *S*-palmitoylation detection method and the acyl-polyethylene glycol (PEG) exchange (APE) assay. An alkyne analog of palmitic acid (PA), 17-octadecynoic acid (17-ODYA), covalently incorporated into MTDH proteins and subsequently enriched by click chemistry and it's indicated that MTDH *S*-palmitoylation (Fig. 1A and Supplemental Fig. S1A). In the APE assay, thioester bonds of the *S*-palmitoylated cysteines are cleaved by hydroxylamine (HAM) and alkylated with methoxy-PEG-maleimide, resulting in a detectable increase in molecular weight of the *S*-palmitoylated substrates. We observed the MTDH band shift in HAM treated group, affirming MTDH *S*-palmitoylation in HEK293T cells and several breast cancer cell lines (Fig. 1B and Supplemental Fig. S1B). To investigate MTDH *S*-palmitoylation level across different cellular components, we isolated the ER, cytoplasm, and nucleus fractions from HEK293T cells respectively. Compared with the whole cell lysates, there is no significant difference in the MTDH *S*-palmitoylation level across different cell fractions by the APE assay (Fig. 1C, D, and Supplemental Fig. S1C). Notably, human MTDH has only a single cysteine residue—Cys-75—located near its transmembrane domain (TMD). To verify Cys-75 is the *S*-palmitoylated site of MTDH, we generated a cysteine to serine mutant (MTDH-CS) and assessed its *S*-palmitoylation by the APE assay. As expected, *S*-palmitoylation of MTDH is completely abolished by this mutation (Fig. 1E).

Furthermore, to determine the main subcellular localization of MTDH and investigate whether *S*-palmitoylation change the MTDH localization, we performed immunofluorescence assay. Interestingly, both SF-tagged (S-protein/FLAG-tagged) MTDH and MTDH-CS mutant are co-localized with calnexin in HeLa cells (Fig. 1F). This indicates MTDH is primarily localized in the ER, regardless of its *S*-palmitoylation status. The inhibitor of 2-bromopalimate (2BP) is commonly used to reduce *S*-palmitoylation level (1). Incubating with the *S*-palmitoylation inhibitor of 2BP, the MTDH consistently localizes in ER (Supplemental Fig. S1D). In addition, the reduction of endogenous MTDH *S*-palmitoylation level was not significant compared with calnexin in HEK293T cells under 100 μM 2BP treatment for 24 h (Supplemental Fig. S1E). To exclude the possibility of MTDH undergoing PATs-independent auto-*S*-palmitoylation, we purified 6×His-tagged MTDH expressed in BL21 (DE3) cells. Contrasted with MTDH expressed in eukaryotes, MTDH expressed in prokaryotes does not undergo *S*-palmitoylation which detected by the acyl-biotin exchange (ABE) assay (Fig. 1G). That implies 2BP is not an appropriate inhibitor to reduce MTDH *S*-palmitoylation. This finding is consistent with the earlier proteomic study, which profiles several direct targets of 2BP (28). Taken together, these results suggest that MTDH undergoes *S*-palmitoylation and the *S*-palmitoylation has not alter MTDH localization in ER.

### The N-terminal loop of MTDH involve in *S*-palmitoylation catalytic process

**Fig. 2:**
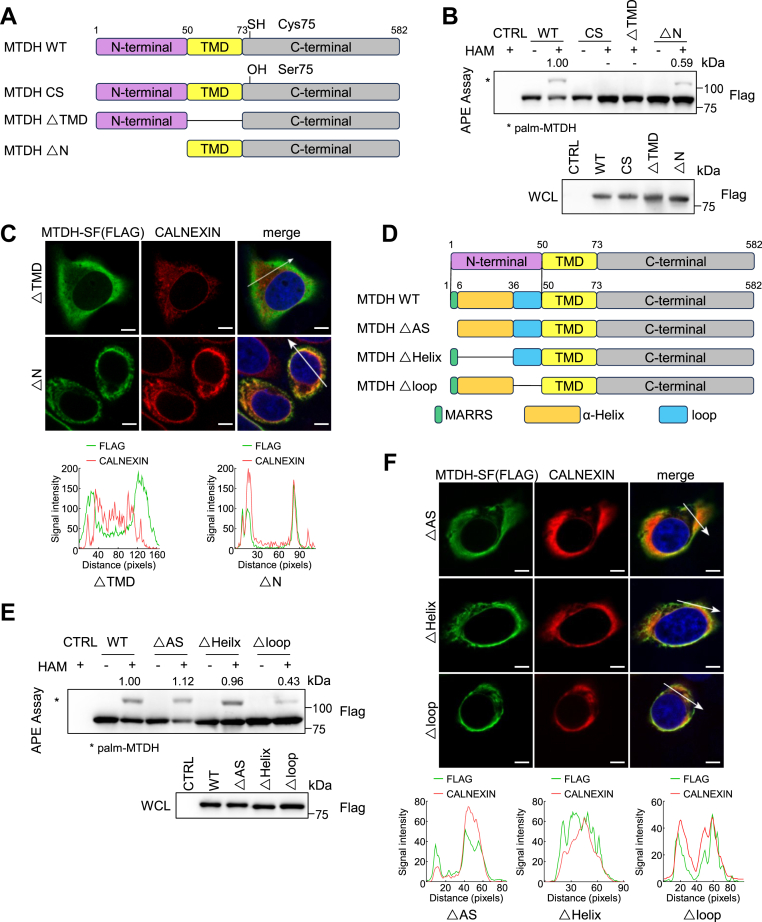
The N-terminal loop of MTDH involve in *S*-palmitoylation catalytic process. A: Schematic representation of MTDH constructs: WT, CS, ΔTMD (transmembrane domain deletion), and ΔN (N-terminal deletion). B: *S*-palmitoylation levels of the above constructs expressed in HEK293T cells, detected by APE assay. Data normalized to WT (+HAM). C: Immunofluorescence of MTDH-ΔTMD or MTDH-ΔN (FLAG) and calnexin in HeLa cells. Scale bar, 5 μm. D: Schematic of additional MTDH deletion mutants: ΔAS (amino acids 2–6), ΔHelix (6–36), and ΔLoop (37–50). E: APE assay analysis of *S*-palmitoylation in MTDH-WT and indicated deletion mutants. Data are normalized to WT (+HAM). F: Immunofluorescence of ΔAS, ΔHelix, or ΔLoop (FLAG) and calnexin in HeLa cells. Scale bar, 5 μm. Results in (B), (C), (E) and (F) were repeated independently at least three times.

During the process of detecting exogenously expressed MTDH *S*-palmitoylation level, we found that the *S*-palmitoylation level of the N-terminal SF-tagged MTDH (SF-MTDH) is significantly decreased than the C-terminal SF-tagged MTDH (MTDH-SF) (Supplemental Fig. S2A). Due to the MTDH TMD region is located near its N-terminus, we first considered whether the N-terminal SF-tag altered the ER localization of MTDH (Fig. 2A). The immunofluorescence results show that SF-MTDH co-localized with calnexin, indicating that the N-terminal SF-tag has not influence the ER localization of MTDH (Supplemental Fig. S2B). Next, to investigate whether the N-terminus of MTDH itself affects its *S*-palmitoylation level, we constructed two MTDH deletion mutants with C-terminal SF-tagged, including deletion of TMD (△TMD), and deletion of N-terminus (△N). As anticipated, *S*-palmitoylation level of △N is considerably lower than the wild-type (WT) MTDH, and *S*-palmitoylation of △TMD is not detected, similar to the MTDH-CS mutant by the APE assay (Fig. 2B). And also, the ER localization of △TMD has basically disappeared, while △N still maintains its ER localization (Fig. 2C). Taken together, these results indicate that MTDH requires ER localization to undergo *S*-palmitoylation, and MTDH N-terminus affects its *S*-palmitoylation level.

Furthermore, to clarify which region of the N-terminus affects the MTDH *S*-palmitoylation level, we conducted structure prediction. According to the AlphaFold2, extra ER domain of MTDH could be divided into three parts: five amino acids Met-Ala-Arg -Arg-Ser (MARRS, green), an α-Helix (orange), and a flexible loop (blue) (Fig. 2D and Supplemental Fig. S2C). We generated another three different MTDH deletion mutants with C-terminal SF-tagged constructs, including deletion of ARRS (△AS), deletion of α-Helix (△Helix), and deletion of loop (△loop). The *S*-palmitoylation level of △loop was significantly decreased than the other two deletion mutants and wildtype MTDH (Fig. 2E). Meanwhile, these mutant form of MTDH maintain the ER localization (Fig. 2F), and the △loop-MTDH with less *S*-palmitoylation level localized ER, which is consistent with the phenomenon of △N-MTDH. These results indicate the loop region in the MTDH N-terminus influences its *S*-palmitoylation level.

Next, we utilized AlphaFold3 to predict the structures of △Helix-MTDH and △loop-MTDH (Supplemental Fig. S2D). The deletion of the N-terminal loop region altered the overall topology of MTDH (Supplemental Fig. S2D), highlighting its crucial role in facilitating *S*-palmitoylation. However, the topology of currently constructed mutants has deviated from that of the SF-MTDH, in which the *S*-palmitoylation reducing phenomenon was initially observed (Supplemental Fig. S2A). Given that the N-terminus of SF-MTDH is prolonged, we duplicated the first ten amino acids of the α-Helix to generate the MTDH-ADD mutant (Supplemental Fig. S2E). MTDH-ADD, which is also localized in the ER, exhibited a slightly reduced *S*-palmitoylation level (Supplemental Fig. S2F, G). According to the AlphaFold3 predicted structures, SF-MTDH and MTDH-ADD have a similar α-Helix length (Supplemental Fig. S2H, I). Taken together, these results suggest that the N-terminal topology of MTDH influences its *S*-palmitoylation without altering ER localization. Specifically, the flexible loop is critical for maintaining MTDH *S*-palmitoylation, while the length of the α-helix also contributes to its regulation.

### ZDHHC1 and ZDHHC9 are the palmitoyl acyltransferases of MTDH

**Fig. 3:**
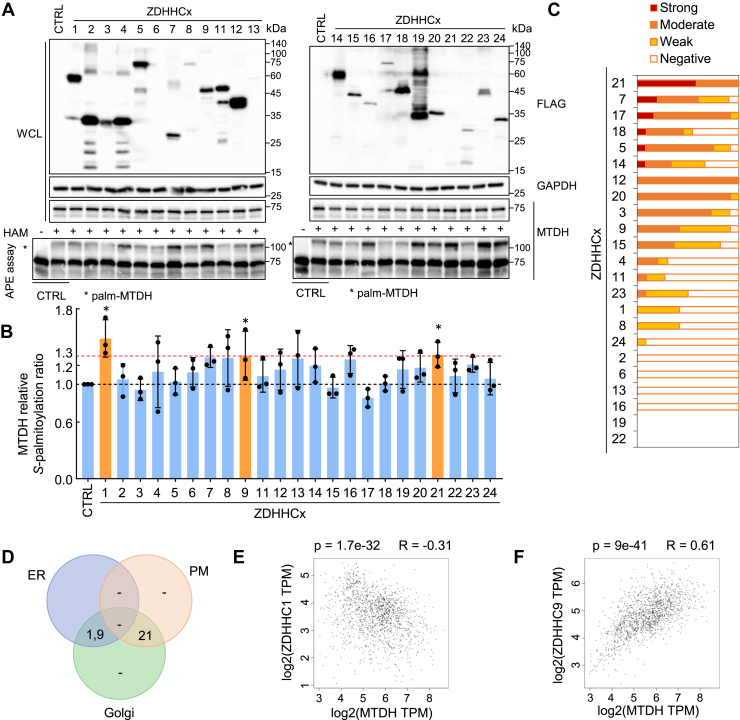
Screening for candidate PATs mediating MTDH *S*-palmitoylation. A: APE assay analysis of endogenous MTDH *S*-palmitoylation in HEK293T cells transfected with individual ZDHHC-SF constructs or empty vector. The experiments were repeated independently at least three times. B: Quantification of (A); ZDHHC candidates promoting MTDH palmitoylation are highlighted in orange. Data normalized to CTRL (+HAM). Statistical analysis by two-tailed Student's *t* test; *P* < 0.05. C: Expression profile of selected ZDHHCs in breast cancer (BRCA) based on Human Protein Atlas IHC data. D: Venn diagram summarizing subcellular localization of ZDHHC family members. E, F: Correlation between MTDH and ZDHHC1 (E) or ZDHHC9 (F) gene expression in TCGA BRCA and GTEx datasets analyzed using GEPIA (Spearman correlation).

To identify the specific PATs catalyzing MTDH *S*-palmitoylation, we overexpressed 23 SF-tagged ZDHHC PATs in HEK293T cells and detected the MTDH *S*-palmitoylation level by performing the APE assay. Among them, ZDHHC1, ZDHHC9, and ZDHHC21 increased MTDH *S*-palmitoylation level by at least 1.3 times (Fig. 3A, B). Next, we aimed to narrow down the potential candidates by integrating the protein expression level in BRCA tissues with subcellular localization information. Expression analysis using the Human Protein Atlas (HPA, https://www.proteinatlas.org/) confirmed that ZDHHC1, ZDHHC9, and ZDHHC21 are all expressed in BRCA tissues (Fig. 3C). Here, we found that the MTDH *S*-palmitoylation is crucial for ER membrane localization. Therefore, we excluded ZDHHC21 from further consideration due to its lack of ER localization, according to Uniprot subcellular localization annotation (https://www.uniprot.org/) (Fig. 3D). Finally, we examined gene expression correlations using the Gene Expression Profiling Interactive Analysis (GEPIA), and found that the MTDH expression is upregulated in BRCA (Supplemental Fig. S3A). Interestingly, ZDHHC1 and ZDHHC9 expression no significant difference between tumor and normal tissues (Supplemental Fig. S3B, C); however, the expression of ZDHHC1 robustly decreased in ER-negative tumor tissues (Supplemental Fig. S3D). MTDH expression was significantly negatively correlated with ZDHHC1 (*P* = 1.7e-32) and positively correlated with ZDHHC9 (*P* = 9e-41) (Fig. 3E, F). Taken together, these results suggest that ZDHHC1 and ZDHHC9 are the most likely PAT candidates mediating MTDH *S*-palmitoylation in BRCA.

**Fig. 4:**
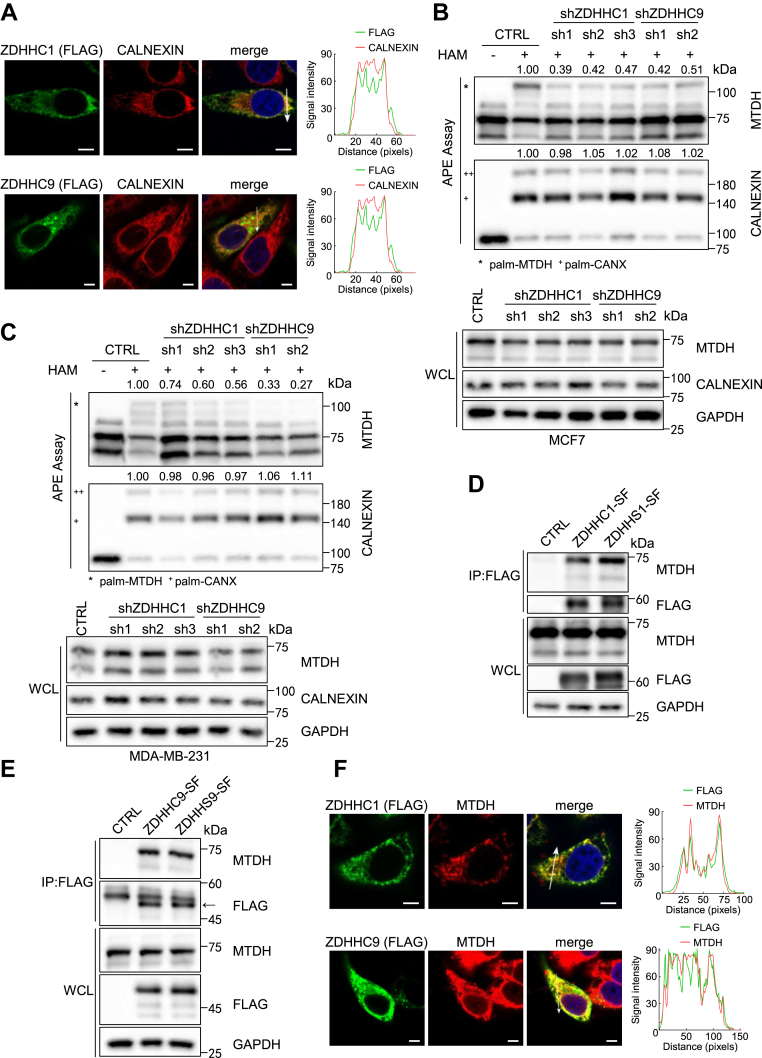
ZDHHC1 and ZDHHC9 catalyze MTDH *S*-palmitoylation. A: Immunofluorescence of ZDHHC1 or ZDHHC9 (FLAG) with calnexin in HeLa cells. Scale bar, 5 μm. B, C: APE assay of MTDH *S*-palmitoylation in MCF7 (B) and MDA-MB-231 (C) cells with ZDHHC1 or ZDHHC9 knockdown. Data normalized to CTRL (+HAM). D, E: Co-immunoprecipitation showing interaction of MTDH with ZDHHC1/ZDHHS1 (D) and ZDHHC9/ZDHHS9 (E) in HEK293T cells. F: Immunofluorescence of ZDHHC1 or ZDHHC9 (FLAG) with MTDH in HeLa cells. Scale bar, 5 μm. All experiments were repeated independently at least three times.

Next, to investigate whether ZDHHC1 and ZDHHC9 regulate MTDH *S*-palmitoylation, we conducted an immunofluorescence assay. Exogenous SF-tagged ZDHHC1 and ZDHHC9 co-localized with calnexin in HeLa cells (Fig. 4A). Next, we performed loss-of-function experiments by knocking down the expression of ZDHHC1 and ZDHHC9 in MCF7 and MDA-MB-231 cells, validated by quantitative real-time PCR (qPCR) (Supplemental Fig. S4A–D). Further, APE assays revealed that endogenous MTDH *S*-palmitoylation level was decreased upon ZDHHC1 or ZDHHC9 knockdown (Fig. 4B, C). In contrast, overexpressed ZDHHC1 and ZDHHC9 augmented endogenous MTDH *S*-palmitoylation level, whereas the catalytically inactive mutants, ZDHHS1 and ZDHHS9, failed to do so (Supplemental Fig. S4E, F).

PATs function can be regulated by *S*-palmitoylation cascades (29). The specific ZDHHC could be *S*-palmitoylated by the other PATs, thereby facilitating the *S*-palmitoylation of its substrates. Therefore, we conducted a pull-down assay and found that SF-tagged ZDHHC1 and ZDHHC9, as well as their inactive mutants form of ZDHHS1 and ZDHHS9, were interacted with endogenous MTDH (Fig. 4D, E). Meanwhile, ZDHHC1 and ZDHHC9 were co-localized with MTDH in ER in HeLa cells (Fig. 4F). Furthermore, coimmunoprecipitation (co-IP) assays revealed that MTDH was detected in the immunoprecipitates of both ZDHHC1 and ZDHHS1, and MTDH pulled down ZDHHC1 and ZDHHS1 (Supplemental Fig. S4G, H). In parallel, ZDHHC9 and ZDHHS9 pulled down MTDH (Supplemental Fig. S4I). However, we did not observe MTDH pull down ZDHHC9 or ZDHHS9 (Supplemental Fig. S4J). These findings suggest that ZDHHC1 and ZDHHC9 potentially interact with MTDH and facilitate its *S*-palmitoylation.

### MTDH is *S*-depalmitoylated by APT1

**Fig. 5:**
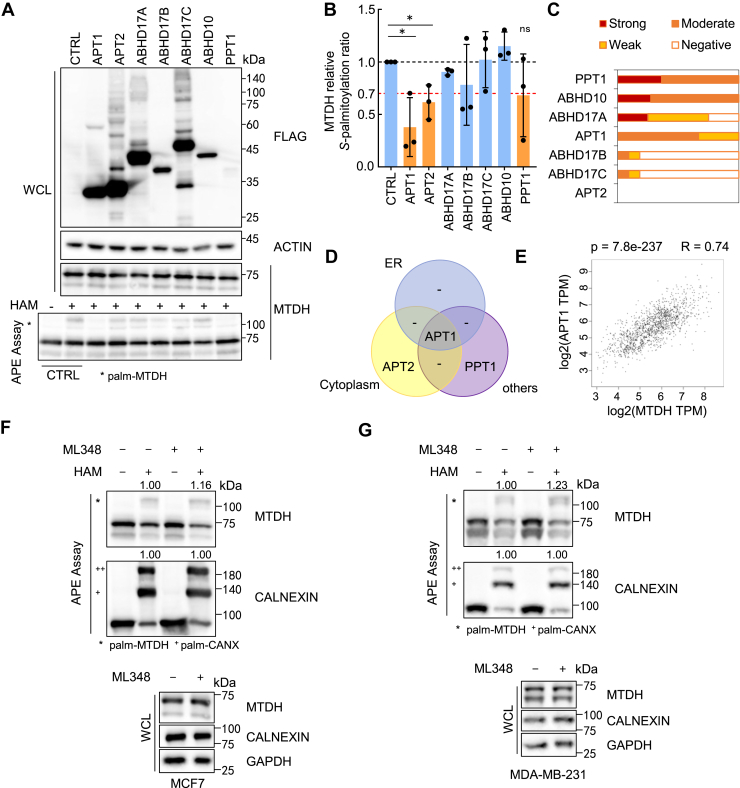
APT1 mediates *S*-depalmitoylation of MTDH. A: APE assay of MTDH *S*-palmitoylation in HEK293T cells transfected with APT-SF constructs or empty vector. B: Quantification of (A); APT1 identified as a major candidate (orange). Data normalized to CTRL (+HAM). Two-tailed Student's *t* test; *P* < 0.05. C: APT expression in BRCA based on Human Protein Atlas IHC data. D: Venn diagram of subcellular localization of APT family members. E: Correlation analysis of MTDH and APT1 gene expression using GEPIA (TCGA BRCA and GTEx). F, G: APE assay of MTDH *S*-palmitoylation in MCF7 (F) or MDA-MB-231 (G) cells treated or untreated with ML348 (15 μM). Data normalized to ML348-untreated (+HAM). Results in (A), (B), (F) and (G) were repeated independently at least three times.

To identify potential enzymes responsible for catalyzing MTDH *S*-depalmitoylation, we transduced most common thioesterases, including APT1/2, ABHD17A/B/C, ABHD10, and PPT1 in HEK293T cells and assessed MTDH *S*-palmitoylation level. Overexpression of APT1 or APT2 reduced MTDH *S*-palmitoylation by approximately 30% (Fig. 5A, B). According to the HPA database, APT1 and PPT1 are expressed in BRCA tissues, while APT2 is undetectable (Fig. 5C), in addition APT1 has ER localization based on the Uniprot database (Fig. 5D). Combined with these results, APT1 is a potential candidate for MTDH *S*-depalmitoylation. Furthermore, GEPIA analysis showed that APT1 expression increased in BRCA (Figure S5A), and revealed a significant positive correlation between MTDH and APT1 expression (*P* = 7.8e-237) (Fig. 5E). In addition, APT1 significantly localized ER (Supplemental Fig. S5B). Taken together, these results suggest that APT1 is the most likely APT candidate mediating MTDH *S*-depalmitoylation in BRCA.

Next, to confirm whether APT1 promote MTDH *S*-depalmitoylation, we treated with 15 μM ML348, a selective APT1 enzyme inhibitor (30) for 12 h in MCF7 and MDA-MB-231 cells, which led to a slight increase in MTDH *S*-palmitoylation level (Fig. 5F, G). Moreover, the pull-down assays showed that APT1 could bind with MTDH, and the immunofluorescence staining also supported that APT1 colocalized with MTDH in the ER (Supplemental Fig. S5C, D). These findings suggest that APT1 may interact with MTDH and facilitate its *S*-depalmitoylation.

### Loss of MTDH *S*-palmitoylation enhances cell migration ability

**Fig. 6:**
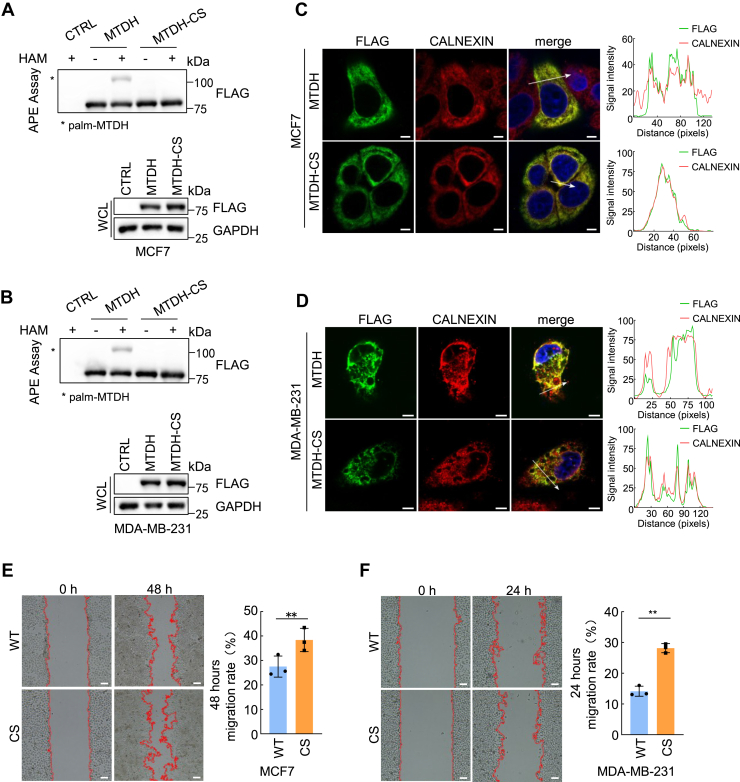
Loss of MTDH *S*-palmitoylation enhances cell migration. A, B: APE assay and immunoblot analysis of MTDH or MTDH-CS in MCF7 (A) and MDA-MB-231 (B) MTDH-knockdown cells rescued with control, WT, or CS constructs. C, D: Immunofluorescence of MTDH-WT or MTDH-CS (FLAG) with calnexin in MCF7 (C) and MDA-MB-231 (D) cells. Scale bar, 5 μm. E, F: Scratch wound assays in MCF7 (E) and MDA-MB-231 (F) MTDH-knockdown cells expressing WT or CS. Migration quantification normalized to WT. Scale bar, 100 μm. Two-tailed Student's *t* test; *P* < 0.01. All experiments were repeated independently at least three times.

It has been shown that MTDH promotes breast cancer metastasis (31). Subsequently, we attempt to understand whether the *S*-palmitoylation of MTDH is relevant for breast cancer cells migration. To achieve this, we firstly generated MTDH knockdown breast cancer cell lines by using shRNAs. Knockdown efficiency was confirmed at both mRNA and protein expression levels in MCF7 and MDA-MB-231 cells (Supplemental Fig. S6A, B). Among them shRNA1 targets the 3′ untranslated region (UTR) of MTDH while shRNA3 targets the coding region (Supplemental Fig. S6C). Next, the FLAG-tagged MTDH-WT or the loss of *S*-palmitoylation mutant form of MTDH-CS transduced into shRNA1 knockdowned MTDH cell lines. The MTDH-WT and MTDH-CS transductions rescued both protein expression levels and *S*-palmitoylation levels (Fig. 6A, B). Immunofluorescence staining supported that MTDH-WT and MTDH-CS all colocalized with calnexin in ER in MCF7 and MDA-MB-231 cells (Fig. 6C, D). These models were then utilized to assess the functional relevance of MTDH *S*-palmitoylation in cell migration. Wound healing assay was performed to detect cell migration. The migration rate of the MTDH *S*-palmitoylation-deficient rescued cell lines was faster than the MTDH-WT rescued cell lines (Fig. 6E, F). Taken together, these findings suggest that the loss of MTDH *S*-palmitoylation enhances the migration ability in breast cancer cells.

### Loss of MTDH *S*-palmitoylation enhances ferroptosis resistance in breast cancer cells

MTDH has been reported to regulate lipid homeostasis, with significantly reduced serum total triglyceride (TG) level observed in MTDH-knockout mice compared to WT-MTDH mice (11). TG is primarily stored in lipid droplets, which could be visualized by BODIPY 493/503 staining (32). Also, we found that the lipid droplet numbers were significantly reduced in MTDH knockdown cells compared with control cells, suggesting that MTDH regulates TG level in breast cancer cells (Supplemental Fig. S7A, B). These results indicate that MTDH contributes to lipid metabolism in breast cancer cells.

**Fig. 7:**
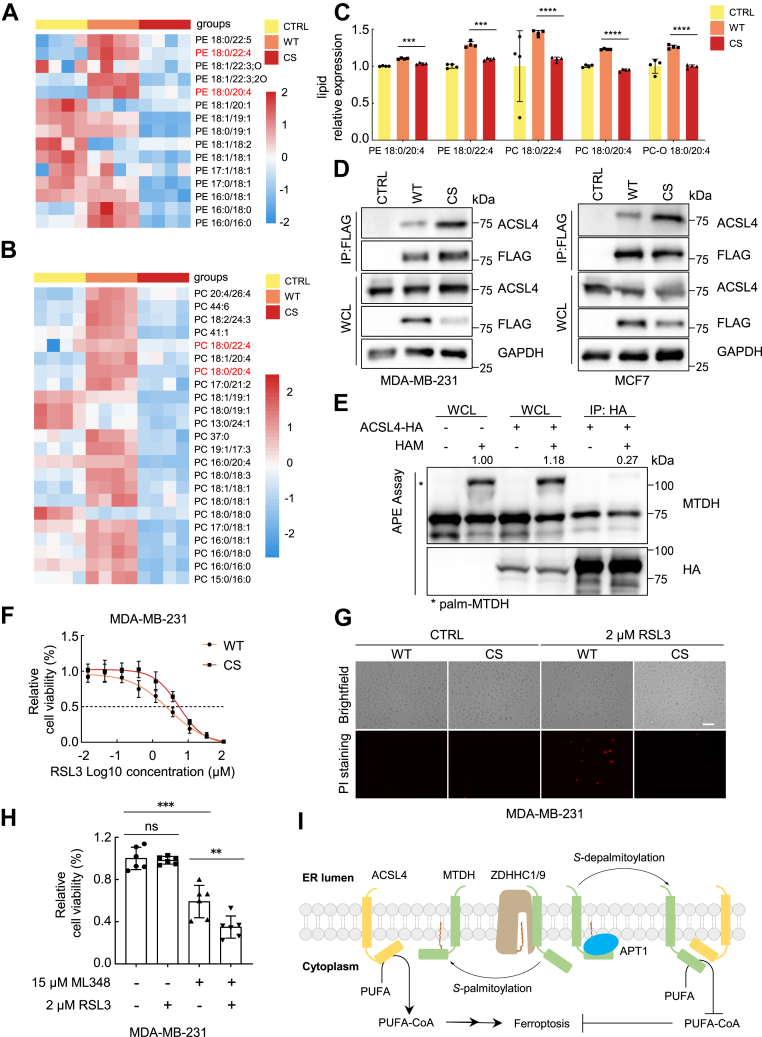
Loss of MTDH *S*-palmitoylation enhances breast cancer cells ferroptosis resistance. A, B: Heatmaps showing major phosphatidylethanolamine (PE) (A) and phosphatidylcholine (PC) (B) species in MTDH-knockdown MDA-MB-231 cells infected with CTRL, WT, or CS constructs. Values normalized to the mean of each species. (n = 4 biological independent samples). C: Quantification of specific PE and PC species in corresponding cells. Data normalized to CTRL. Two-tailed Student's *t* test; ∗∗∗*P* < 0.001, ∗∗∗∗*P* < 0.0001. (n = 4 biological independent samples). D: Co-immunoprecipitation showing interaction between MTDH or MTDH-CS and endogenous ACSL4 in MCF7 and MDA-MB-231 MTDH-knockdown cells. E: APE assay showing *S*-palmitoylation of MTDH pulled down by ACSL4 in HEK293T cells transfected with ACSL4-HA or control. Data normalized to ACSL4–/HAM + group. F: Cell viability analysis of MTDH-knockdown MDA-MB-231 cells expressing WT or CS, treated with RSL3 for 48 h. G: Images of MTDH-knockdown MDA-MB-231 cells expressing WT or CS, treated with RSL3 for 24 h in the presence of PI. H: Cell viability analysis of MDA-MB-231 cells, treated with or without ML348 or RSL3 for 24 h. Data normalized to the group without ML348 and RSL3. Two-tailed Student's *t* test; ∗∗*P* < 0.01, ∗∗∗*P* < 0.001. (n = 3 biological independent samples). I: Schematic model depicting how MTDH *S*-palmitoylation regulates ferroptosis in breast cancer cells. Results in (D), (E), (F), (G) and (H) were repeated independently at least three times.

Accordingly, we next investigated whether the *S*-palmitoylation of MTDH also affects lipid homeostasis. To achieve this, we performed untargeted lipidomic profiling in MDA-MB-231 cell lines, including MTDH-knockdown cells (CTRL), MTDH-WT rescued cells (WT), and MTDH-CS rescued cells (CS). Principal component analysis revealed distinct lipidomic profiles among the three groups (Supplemental Fig. S7C). Similar to the fluorescent staining data of lipid droplets, TG level was elevated in MTDH-WT rescued cells (Supplemental Fig. S6D). However, in MTDH-CS rescued cells, TG level was also increased but remained below that in MTDH-WT rescued cells (Supplemental Fig. S7D, E). Additionally, we noticed the levels of phosphatidylethanolamines (PE) and phosphatidylcholines (PC) were downregulated in the MTDH-CS rescued cells, compared to the MTDH-WT rescued cells (Fig. 7A, B). Taken together, these findings suggest that *S*-palmitoylation of MTDH plays a critical role in regulating lipid composition in breast cancer cells, particularly affecting levels of TG, PC, and PE.

Furthermore, the PC and PE containing C20:4 and C22:4 acyl chains were reduced in the MTDH-CS rescued cells, compared to the MTDH-WT rescued cells (Fig. 7C). Metabolic enzyme of ACSL4 which is localized in ER, promotes the PUFAs to acyl-CoA, especially arachidonic acid (C20:4) and adrenic acid (C22:4) (33, 34). These data indicate that the *S*-palmitoylation of MTDH may affect ACSL4 activity in breast cancer cells. Therefore, to investigate whether MTDH interacts with ACSL4, we performed pull-down assay and observed that HA-tagged ACSL4 interact with endogenous MTDH (Supplemental Fig. S7F). Next, both MTDH-WT and MTDH-CS pulled downed ACSL4, and MTDH-CS had a stronger interaction with ACSL4 in MDA-MB-231 and MCF7 cell lines (Fig. 7D). Considering that MTDH exists in both *S*-palmitoylated and *S*-depalmitoylated states, we hypothesize that ACSL4 preferentially interacts with the *S*-depalmitoylated form of MTDH. To verify this hypothesis, we eluted the immunoprecipitates of ACSL4 and assessed the APE assay to examine the MTDH *S*-palmitoylation level. Overexpression of ACSL4 had no impact on MTDH undergoing *S*-palmitoylation, and the pulled down MTDH by ACSL4 were mainly *S*-depalmitoylated states (Fig. 7E). Taken together, these results suggest that ACSL4 predominantly interacts with *S*-depalmitoylated MTDH, and this interaction may affect ACSL4 activity.

Remodeling of glycerophospholipids (including PC and PE) in the cell membrane is one of the ferroptosis hallmarks (14). ACSL4 is a key lipid metabolism enzyme in ferroptosis (35). We first employed the Cancer Treatment Response Portal (CTRP) database (https://portals.broadinstitute.org/ctrp.v2.1/) to investigate the correlation between MTDH expression and ferroptosis, found that high MTDH expression level correlated with enhanced resistance to ferroptosis (Supplemental Fig. S7G). Beside a previous study also supports this notion, showing that MTDH enhances the sensitivity of breast cancer cells to ferroptosis (36). Based on this, we considered whether *S*-palmitoylation of MTDH influence ferroptosis sensitivity in breast cancer cells. Consequently, MTDH-WT and MTDH-CS rescued cells were stimulated with varying concentrations of RSL3, which is a commonly used ferroptosis agonist (37) by directly targeting GPX4, revealing that MTDH-CS rescued MDA-MB-231 cells exhibited reduced sensitivity to ferroptosis (Fig. 7F). However, this was not observed in rescued MCF7 cells (Supplemental Fig. S7H). This may be attributed to the MCF7 cells intrinsic insensitivity to RSL3 (Supplemental Fig. S7I). In parallel, MTDH-CS rescued MDA-MB-231 or MCF7 cells that almost completely resisted RSL3-induced cell death, indicated by staining with propidium iodide (PI) (Fig. 7G and Supplemental Fig. S7J). Furthermore, to investigate the effect of increasing MTDH *S*-palmitoylation level on ferroptosis, we treated MDA-MB-231 or MCF7 with RSL3 and ML348 together. As expected, RSL3 alone reduced cell viability, whereas the addition of ML348 further sensitized breast cancer cells to RSL3-induced ferroptosis. ML348 itself showed no significant effect on cell viability (Fig. 7H and Supplemental Fig. S7K). Taken together, these findings suggest that *S*-palmitoylation of MTDH enhances ferroptosis sensitivity in breast cancer cells, may particularly in MDA-MB-231, but not in estrogen receptor-positive (ER+) MCF7 cells.

## Discussion

*S*-palmitoylation is the only reversible lipidation that plays a crucial role in protein conformation, localization, stability, and protein-protein interaction, thereby influencing a wide range of physiological and pathological processes, including cancer progression (3, 38, 39, 40, 41). Although *S*-palmitoylation of MTDH has been overexpressed in early proteomic studies, its functional implications in cancer progression have remained largely unexplored (6, 7). In the present study, we revealed that *S*-palmitoylation of MTDH negatively regulates its interaction with ACSL4, thereby modulating the ferroptosis sensitivity in breast cancer cells. Specifically, the *S*-palmitoylation of MTDH is dynamically catalyzed at Cys-75 by ZDHHC1/9 and APT1, functioning in a reversible manner (Fig. 7I).

*S*-palmitoylation is catalyzed by the ZDHHC family via the ping-pong mechanism (1). In our study, we found ZDHHC1 and ZDHHC9 *S*-palmitoylated MTDH (Fig. 4). However, ZDHHC6 is also reported to catalyze MTDH *S*-palmitoylation in HCC cells (8). This phenomenon of multiple ZDHHCs can be identified to *S*-palmitoylate the same substrate is not rare. Similar phenomenon has been observed with other proteins, such as nucleotide oligomerization domain-like receptor protein 3 (NLRP3) (42, 43, 44, 45), Gasdermin D (GSDMD) (46, 47, 48), and GPX4 (15, 16). Moreover, distinct substrates have been identified for the same ZDHHC across different cell lines at the proteomics level (49). Together, these reflects PAT redundancy and the lack of clearly defined recognition sequences, complicating the elucidation of ZDHHC-substrate network. A comprehensive view of the ZDHHC-substrate network needs to be constructed to clearly understand regulatory mechanisms of *S*-palmitoylation. Furthermore, we found N-terminal of MTDH affect its *S*-palmitoylation level, indicating the topology of substrates influences the catalyze process (Fig. 2). Additionally, we found 2BP is not useful to inhibit MTDH *S*-palmitoylation level (Supplemental Fig. S1C–E), which indicate that 2BP has nonselective off-target effects. Therefore, more specific *S*-palmitoylation inhibitors has to be developed (1, 50).

Energy metabolism reprogramming is one hallmarks of cancer (51). MTDH has been reported to regulate glucose metabolism and lipid metabolism in cancer cells (11, 52, 53). In our study, we found that *S*-palmitoylation of MTDH regulates lipid metabolism particularly affecting PC, PE, and TG (Fig. 7 and Supplemental Fig. S7). Lipid metabolism disorders can modulate cellular ferroptosis (54). Iron metabolism, frequently disrupted in cancer progression, is often characterized by iron overload in in BRCA (55, 56, 57). Ferroptosis, an iron-dependent PCD, is considered as a potential novel therapeutic target of BRCA (58, 59). ACSL4 serves as a key regulator in ferroptosis by promoting the accumulation of PUFA-PLs in the cell membrane (60). We demonstrated that the loss of *S*-palmitoylation on MTDH enhances its interaction with ACSL4 and subsequently reduces ferroptosis sensitivity in MDA-MB-231 cells (Fig. 7). Interestingly, this effect was not fully evident in MCF7 cells, even though the *S*-palmitoylation-deficient mutant of MTDH similarly showed increased binding to ACSL4 (Fig. 7 and Supplemental Fig. S7). Since the experiments were performed in MTDH-knockdown cell lines, the residual baseline of MTDH *S*-palmitoylation level in MCF7 cells might have been sufficiently high to mask the positive effects. Furthermore, MCF7 is an ER + breast cancer cell lines, and ER + breast cancers are known to suppress ferroptosis sensitivity (61). These findings highlight a cell type-specific regulatory mechanism of ferroptosis mediated by MTDH *S*-palmitoylation.

Inhibiting GPX4 is a more direct way to suppress tumor progression by inducing ferroptosis (62). But traditional GPX4 inhibitors, such as RSL3, also target immune cells, impairing their antitumor ability in vivo (63, 64). MTDH is essential for mammary tumorigenesis but dispensable for normal development (65). Therefore, it is reasonable to infer that altering the *S*-palmitoylation level of MTDH would have minimal impact on the normal physiological state of cells. Previous studies have reported that reducing *S*-palmitoylation levels, either directly or indirectly, enhances the sensitivity of tumor cells to ferroptosis (15, 16, 17, 18, 19, 20). However, selectively inhibiting specific palmitoyl acyltransferases (PATs) remains challenging (1). In our study, we found that ML348, a specific inhibitor of APT1, significantly enhanced RSL3-induced ferroptosis sensitivity in breast cancer cells (Fig. 7 and Supplemental Fig. S7). Therefore, targeting MTDH *S*-palmitoylation to enhance ferroptosis sensitivity may represent a novel cancer therapeutic strategy.

Additionally, an early report indicates that MTDH *S*-palmitoylation suppresses its interaction with SND1, thereby inhibiting tumor growth of HCC (8). From the mechanistic perspective, it is similar to our findings that MTDH *S*-palmitoylation inhibits its interaction with certain proteins, including ACSL4 and SND1. Disrupting MTDH-SND1 complex makes contribution to BRCA treatment (31, 66). Collectively, our results highlight the crucial role of MTDH *S*-palmitoylation in modulating protein interactions and ferroptosis sensitivity, suggesting that inhibition of MTDH *S*-palmitoylation may be a potential strategy for breast cancer therapy.

## Data Availability

All data reported in this paper will be shared by the lead contact upon request.

This paper does not report original code.

Any additional information required to reanalyze the data reported in this paper is available from the lead contact upon request.

## Supplemental Data

This article contains supplemental data.

## Conflict of Interest

The authors declare that they do not have any conflicts of interest with the content of this article.

## Supplemental Data

Supplementary Figures

Key Resource Table

## References

[1] Pei S.J., Piao H.L. (2024). Exploring protein *S*-Palmitoylation: Mechanisms, detection, and strategies for inhibitor discovery. ACS Chem. Biol..

[2] Mesquita F.S., Abrami L., Samurkas A., van Der Goot F.G. (2023). *S*-acylation: an orchestrator of the life cycle and function of membrane proteins. FEBS J..

[3] Mesquita F.S., Abrami L., Linder M.E., Bamji S.X., Dickinson B.C., van der Goot F.G. (2024). Mechanisms and functions of protein *S*-acylation. Nat. Rev. Mol. Cell Biol..

[4] Rinschen M.M., Ivanisevic J., Giera M., Siuzdak G. (2019). Identification of bioactive metabolites using activity metabolomics. Nat. Rev. Mol. Cell Biol..

[5] Wang W., Liu X., Chen H., Ling T., Xia T., Liu X. (2020). Cholesterol as a functional metabolite cooperates with metadherin in cancer cells. Chin. Chem. Lett..

[6] Martin B.R., Cravatt B.F. (2009). Large-scale profiling of protein palmitoylation in mammalian cells. Nat. Methods.

[7] Martin B.R., Wang C., Adibekian A., Tully S.E., Cravatt B.F. (2011). Global profiling of dynamic protein palmitoylation. Nat. Methods.

[8] Zhou B., Wang Y., Zhang L., Shi X., Kong H., Zhang M. (2022). The palmitoylation of AEG-1 dynamically modulates the progression of hepatocellular carcinoma. Theranostics.

[9] Lee S.G., Kang D.C., DeSalle R., Sarkar D., Fisher P.B., Sarkar D., Fisher P.B. (2013). Aeg-1/Mtdh/Lyric Implicated in Multiple Human Cancers.

[10] Hu G., Chong R.A., Yang Q., Wei Y., Blanco M.A., Li F. (2009). MTDH activation by 8q22 genomic gain promotes chemoresistance and metastasis of poor-prognosis breast cancer. Cancer Cell.

[11] Robertson C.L., Srivastava J., Siddiq A., Gredler R., Emdad L., Rajasekaran D. (2015). Astrocyte Elevated Gene-1 (AEG-1) regulates lipid homeostasis. J. Biol. Chem..

[12] Yuan J.Y., Ofengeim D. (2024). A guide to cell death pathways. Nat. Rev. Mol. Cell Biol..

[13] Chen X., Li J.B., Kang R., Klionsky D.J., Tang D.L. (2021). Ferroptosis: machinery and regulation. Autophagy.

[14] Lee J., Roh J.L. (2025). Lipid metabolism in ferroptosis: unraveling key mechanisms and therapeutic potential in cancer. Biochim. Biophys. Acta-Reviews on Cancer.

[15] Zhou L., Lian G., Zhou T., Cai Z., Yang S., Li W. (2025). Palmitoylation of GPX4 via the targetable ZDHHC8 determines ferroptosis sensitivity and antitumor immunity. Nat. Cancer.

[16] Huang B., Wang H., Liu S., Hao M., Luo D., Zhou Y. (2025). Palmitoylation-dependent regulation of GPX4 suppresses ferroptosis. Nat. Commun..

[17] Wang Z., Wang Y., Shen N., Liu Y., Xu X., Zhu R. (2024). AMPKα1-mediated ZDHHC8 phosphorylation promotes the palmitoylation of SLC7A11 to facilitate ferroptosis resistance in glioblastoma. Cancer Letters.

[18] Shi Z., Li Z., Jin B., Ye W., Wang L., Zhang S. (2023). Loss of LncRNA DUXAP8 synergistically enhanced sorafenib induced ferroptosis in hepatocellular carcinoma via SLC7A11 de-palmitoylation. Clin. Transl. Med..

[19] Qian Z.W., Jiang Y., Cai Y., Gao E.R., Wang C.Z., Dong J.F. (2025). FASN inhibits ferroptosis in breast cancer via USP5 palmitoylation-dependent regulation of GPX4 deubiquitination. J. Exp. Clin. Cancer Res..

[20] Shao S., Li W., Hong Y., Zeng R., Zhu L., Yi L. (2025). ZDHHC2-Dependent palmitoylation dictates ferroptosis and castration sensitivity in prostate cancer via controlling ACSL4 degradation and lipid peroxidation. Adv. Sci..

[21] Drisdel R.C., Green W.N. (2004). Labeling and quantifying sites of protein palmitoylation. Biotechniques..

[22] Percher A., Ramakrishnan S., Thinon E., Yuan X., Yount J.S., Hang H.C. (2016). Mass-tag labeling reveals site-specific and endogenous levels of protein S-fatty acylation. Proc Natl Acad Sci U. S. A..

[23] Stirling D.R., Swain-Bowden M.J., Lucas A.M., Carpenter A.E., Cimini B.A., Goodman A. (2021). CellProfiler 4: improvements in speed, utility and usability. BMC Bioinf.

[24] Ning Z., Guo X., Liu X., Lu C., Wang A., Wang X. (2022). USP22 regulates lipidome accumulation by stabilizing PPARγ in hepatocellular carcinoma. Nat. Commun..

[25] Cai Z.R., Wang W., Chen D., Chen H.J., Hu Y., Luo X.J. (2024). Diagnosis and prognosis prediction of gastric cancer by high-performance serum lipidome fingerprints. EMBO Mol. Med..

[26] Otkur W., Zhang Y., Li Y., Bao W., Feng T., Wu B. (2024). Spatial multi-omics characterizes GPR35-relevant lipid metabolism signatures across liver zonation in MASLD. Life Metab..

[27] Tsugawa H., Ikeda K., Takahashi M., Satoh A., Mori Y., Uchino H. (2020). A lipidome atlas in MS-DIAL 4. Nat. Biotechnol..

[28] Davda D., El Azzouny M.A., Tom C.T., Hernandez J.L., Majmudar J.D., Kennedy R.T. (2013). Profiling targets of the irreversible palmitoylation inhibitor 2-bromopalmitate. ACS Chem. Biol..

[29] Anwar M.U., van der Goot F.G. (2023). Refining *S*-acylation: structure, regulation, dynamics, and therapeutic implications. J. Cell Biol..

[30] Won S.J., Cheung See Kit M., Martin B.R. (2018). Protein depalmitoylases. Crit. Rev. Biochem. Mol. Biol..

[31] Shen M., Wei Y., Kim H., Wan L., Jiang Y.Z., Hang X. (2022). Small-molecule inhibitors that disrupt the MTDH-SND1 complex suppress breast cancer progression and metastasis. Nat. Cancer.

[32] Olzmann J.A., Carvalho P. (2019). Dynamics and functions of lipid droplets. Nat. Rev. Mol. Cell Biol..

[33] Kagan V.E., Mao G.W., Qu F., Angeli J.P.F., Doll S., St Croix C. (2017). Oxidized arachidonic and adrenic PEs navigate cells to ferroptosis. Nat. Chem. Biol..

[34] Radif Y., Ndiaye H., Kalantzi V., Jacobs R., Hall A., Minogue S. (2018). The endogenous subcellular localisations of the long chain fatty acid-activating enzymes ACSL3 and ACSL4 in sarcoma and breast cancer cells. Mol. Cell. Biochem..

[35] Doll S., Proneth B., Tyurina Y.Y., Panzilius E., Kobayashi S., IngoId I. (2017). ACSL4 dictates ferroptosis sensitivity by shaping cellular lipid composition. Nat. Chem. Biol..

[36] Bi J., Yang S., Li L., Dai Q., Borcherding N., Wagner B.A. (2019). Metadherin enhances vulnerability of cancer cells to ferroptosis. Cell Death Dis..

[37] Yang W.S., SriRamaratnam R., Welsch M.E., Shimada K., Skouta R., Viswanathan V.S. (2014). Regulation of ferroptotic cancer cell death by GPX4. Cell.

[38] Chen B., Sun Y., Niu J., Jarugumilli G.K., Wu X. (2018). Protein lipidation in cell signaling and diseases: function, regulation, and therapeutic opportunities. Cell Chem Biol.

[39] Ko P.J., Dixon S.J. (2018). Protein palmitoylation and cancer. EMBO Rep.

[40] Zhou B.H., Hao Q.Y., Liang Y.M., Kong E.Y. (2023). Protein palmitoylation in cancer: molecular functions and therapeutic potential. Mol. Oncol..

[41] Tate E.W., Soday L., de la Lastra A.L., Wang M., Lin H.N. (2024). Protein lipidation in cancer: mechanisms, dysregulation and emerging drug targets. Nat. Rev. Cancer..

[42] Yu T., Hou D., Zhao J.Q., Lu X., Greentree W.K., Zhao Q. (2024). NLRP3 Cys126 palmitoylation by ZDHHC7 promotes inflammasome activation. Cell Rep..

[43] Nie L., Fei C.J., Fan Y.Z., Dang F.B., Zhao Z.Y., Zhu T.F. (2024). Consecutive palmitoylation and phosphorylation orchestrates NLRP3 membrane trafficking and inflammasome activation. Mol. Cell..

[44] Wang L., Cai J., Zhao X., Ma L., Zeng P., Zhou L. (2023). Palmitoylation prevents sustained inflammation by limiting NLRP3 inflammasome activation through chaperone-mediated autophagy. Mol. Cell..

[45] Zheng S.H., Que X.Y., Wang S.X., Zhou Q., Xing X.K., Chen L. (2023). ZDHHC5-mediated NLRP3 palmitoylation promotes NLRP3-NEK7 interaction and inflammasome activation. Mol. Cell..

[46] Du G., Healy L.B., David L., Walker C., El-Baba T.J., Lutomski C.A. (2024). ROS-dependent S-palmitoylation activates cleaved and intact gasdermin D. Nature.

[47] Zhang N., Zhang J., Yang Y., Shan H., Hou S., Fang H. (2024). A palmitoylation-depalmitoylation relay spatiotemporally controls GSDMD activation in pyroptosis. Nat. Cell Biol..

[48] Balasubramanian A., Hsu A.Y., Ghimire L., Tahir M., Devant P., Fontana P. (2024). The palmitoylation of gasdermin D directs its membrane translocation and pore formation during pyroptosis. Sci. Immunol..

[49] Ocasio C.A., Baggelaar M.P., Sipthorp J., de la Lastra A.L., Tavares M., Volaric J. (2024). A palmitoyl transferase chemical-genetic system to map ZDHHC-specific *S*-acylation. Nat. Biotechnol..

[50] Azizi S.-A., Qiu T., Brookes N.E., Dickinson B.C. (2023). Regulation of ERK2 activity by dynamic *S*-acylation. Cell Rep..

[51] Hanahan D., Weinberg R.A. (2011). Hallmarks of cancer: the next generation. Cell.

[52] Bhutia S.K., Kegelman T.P., Das S.K., Azab B., Su Z.Z., Lee S.G. (2010). Astrocyte elevated gene-1 induces protective autophagy. Proc. Natl. Acad. Sci. U. S. A.

[53] Yoo B.K., Emdad L., Su Z., Villanueva A., Chiang D.Y., Mukhopadhyay N.D. (2009). Astrocyte elevated gene-1 regulates hepatocellular carcinoma development and progression. J. Clin. Invest..

[54] Lee H., Horbath A., Kondiparthi L., Meena J.K., Lei G., Dasgupta S. (2024). Cell cycle arrest induces lipid droplet formation and confers ferroptosis resistance. Nat. Commun..

[55] Yu X., Cheng L., Liu S., Wang M., Zhang H., Wang X. (2024). Correlation between ferroptosis and adriamycin resistance in breast cancer regulated by transferrin receptor and its molecular mechanism. FASEB J..

[56] Pinnix Z.K., Miller L.D., Wang W., D'Agostino R., Kute T., Willingham M.C. (2010). Ferroportin and iron regulation in breast cancer progression and prognosis. Sci. Transl. Med..

[57] Roemhild K., von Maltzahn F., Weiskirchen R., Knüchel R., von Stillfried S., Lammers T. (2021). Iron metabolism: pathophysiology and pharmacology. Trends Pharmacol. Sci..

[58] Dixon S.J., Lemberg K.M., Lamprecht M.R., Skouta R., Zaitsev E.M., Gleason C.E. (2012). Ferroptosis: an iron-dependent form of nonapoptotic cell death. Cell.

[59] Su G.H., Xiao Y., You C., Zheng R.C., Zhao S., Sun S.Y. (2023). Radiogenomic-based multiomic analysis reveals imaging intratumor heterogeneity phenotypes and therapeutic targets. Sci. Adv..

[60] Wang Y.Q., Hu M.Z., Cao J., Wang F.X., Han J.R., Wu T.W. (2025). ACSL4 and polyunsaturated lipids support metastatic extravasation and colonization. Cell.

[61] Liang D.G., Feng Y., Zandkarimi F., Wang H., Zhang Z.D., Kim J. (2023). Ferroptosis surveillance independent of GPX4 and differentially regulated by sex hormones. Cell.

[62] Hangauer M.J., Viswanathan V.S., Ryan M.J., Bole D., Eaton J.K., Matov A. (2017). Drug-tolerant persister cancer cells are vulnerable to GPX4 inhibition. Nature.

[63] Ma X., Xiao L., Liu L., Ye L., Su P., Bi E. (2021). CD36-mediated ferroptosis dampens intratumoral CD8+ T cell effector function and impairs their antitumor ability. Cell Metab..

[64] Kim R., Hashimoto A., Markosyan N., Tyurin V.A., Tyurina Y.Y., Kar G. (2022). Ferroptosis of tumour neutrophils causes immune suppression in cancer. Nature.

[65] Wan L., Lu X., Yuan S., Wei Y., Guo F., Shen M. (2014). MTDH-SND1 interaction is crucial for expansion and activity of tumor-initiating cells in diverse oncogene- and carcinogen-induced mammary tumors. Cancer Cell.

[66] Shen M., Smith H.A., Wei Y., Jiang Y.Z., Zhao S., Wang N. (2022). Pharmacological disruption of the MTDH-SND1 complex enhances tumor antigen presentation and synergizes with anti-PD-1 therapy in metastatic breast cancer. Nat. Cancer.

