# Supplementary material for: S-palmitoylation of MTDH regulates ferroptosis resistance in breast cancer cell
Source: J Lipid Res. 2025 Nov 28;67(1):100953. doi: 10.1016/j.jlr.2025.100953 (PMC12794514; doi:10.1016/j.jlr.2025.100953)
Supplement: Supplementary Figures [file mmc1.docx]

**Supplementary Figures**

***S*-palmitoylation of MTDH regulates ferroptosis resistance in breast cancer cell**

**Shaojun Pei^1,5^, Wen Wang^1,4^, Tingze Feng^1^, Qiuping Wang^1^, Yuhan Wang^1^, Hong-Xu Liu^2^, Xinmiao Liang^1,5^ and Hai-long Piao^1,2,3,5,*^**

^1^State Key Laboratory of Phytochemistry and Natural Medicines, Dalian Institute of Chemical Physics, Chinese Academy of Sciences, Dalian 116023, China.

^2^Department of Thoracic Surgery, Cancer Hospital of Dalian University of Technology, Liaoning Cancer Hospital & Institute, Shenyang 110042, China.

^3^Department of Biochemistry & Molecular Biology, School of Life Sciences, China Medical University, Shenyang, 110122, China.

^4^Department of Neurology, The First Affiliated Hospital of Anhui Medical University, Hefei,Anhui,230022，China

^5^University of Chinese Academy of Sciences, Beijing 100049, China.

*Correspondence:

Hai-long Piao, hpiao@dicp.ac.cn, Tel: +86-411-39787236, Fax: 86-411-39787236

**
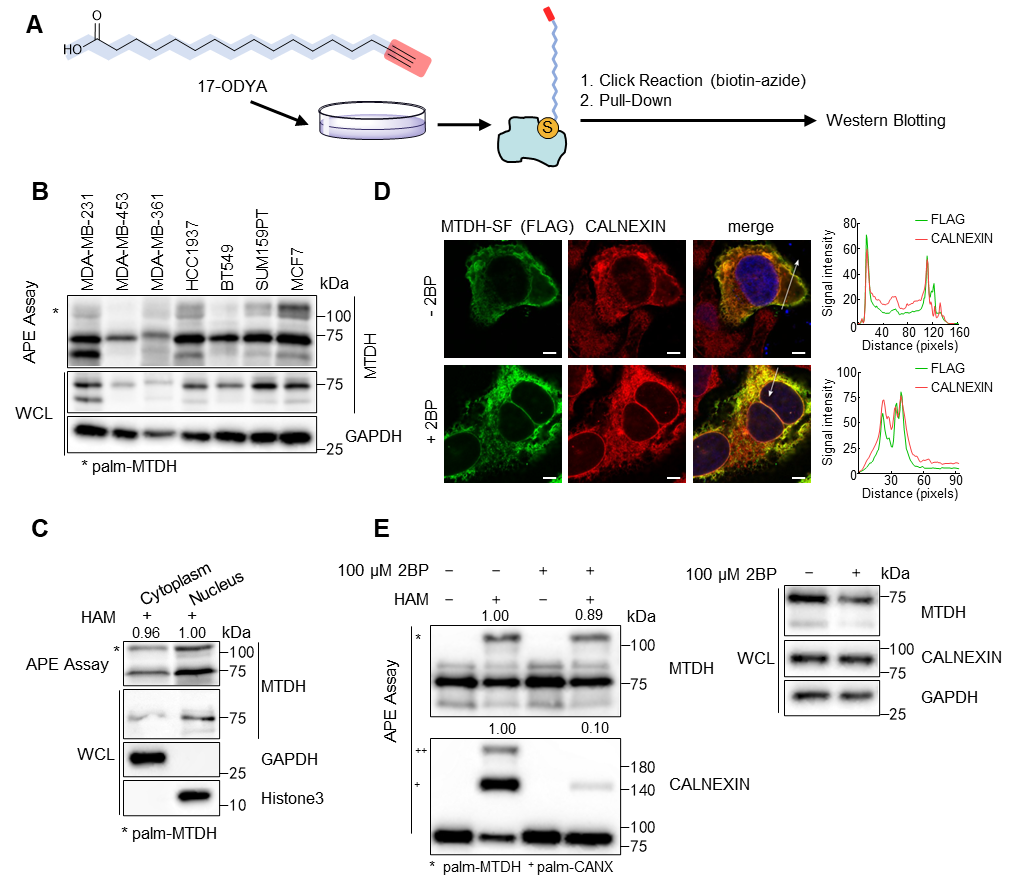
**

**Figure S1. MTDH is *S*-palmitoylated on the endoplasmic reticulum**

(A) Schematic illustration of 17-ODYA labeling and the workflow for detecting *S*-palmitoylated proteins.

(B) APE assay of endogenous MTDH *S*-palmitoylation in MDA-MB-231, MDA-MB-453, MDA-MB-361, HCC1937, BT549, SUM159PT, and MCF7 cells.

(C) APE assay comparing MTDH *S*-palmitoylation in nuclear/cytosolic fractions and whole-cell lysates (WCL) in HEK293FT cells. Data are normalized to WCL.

(D) Immunofluorescence staining of MTDH-SF and calnexin in HeLa cells treated with or without 100 μM 2BP for 24 h. Scale bar, 5 μm.

(E) APE assay of MTDH *S*-palmitoylation in HEK293T cells treated with or without 100 μM 2BP. HAM+: with HAM; HAM–: without HAM. Data are normalized to untreated HAM+ group.

All experiments were independently repeated at least three times.


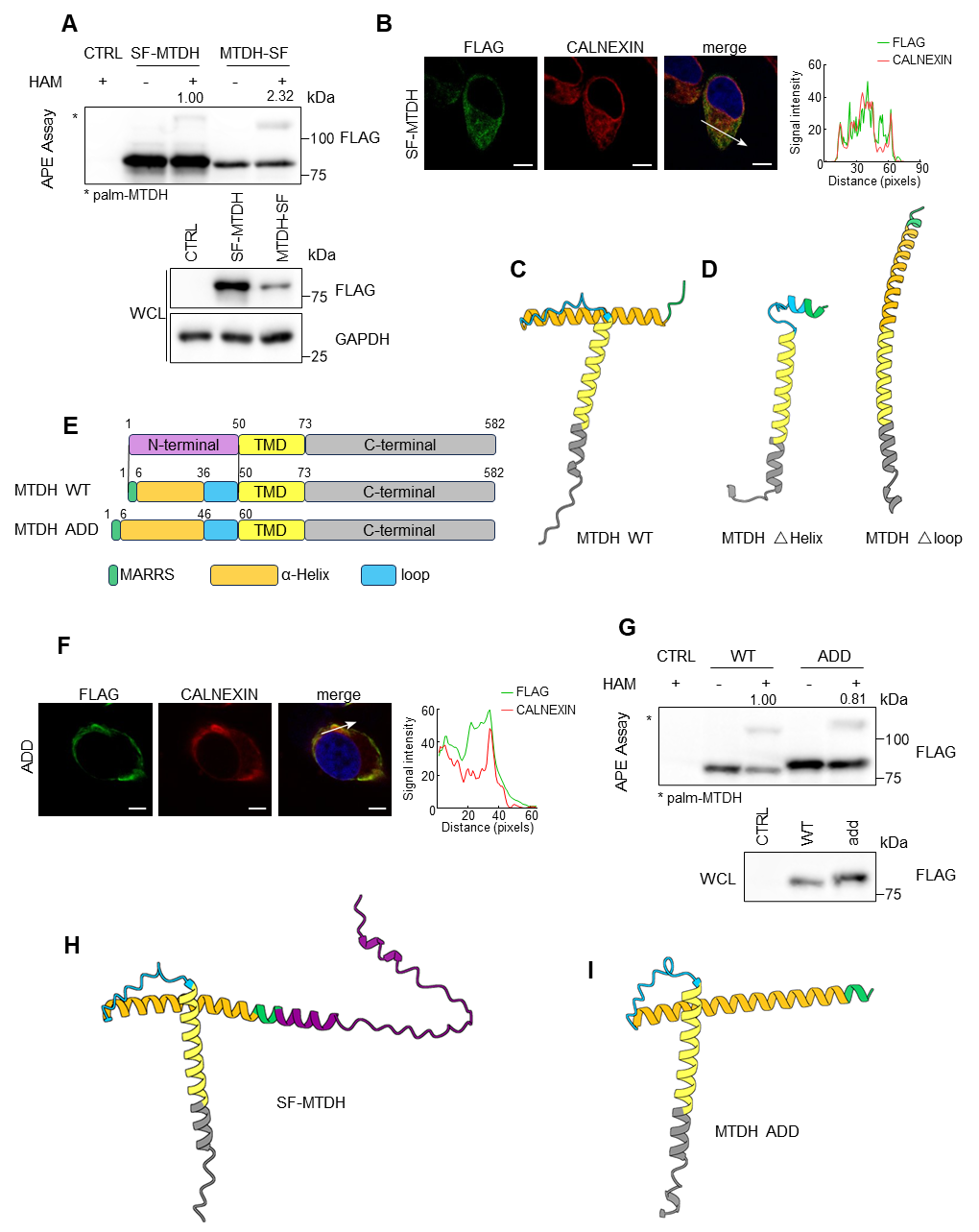


**Figure S2. The N-terminus of MTDH may contribute to its *S*-palmitoylation.**

(A) APE assay of *S*-palmitoylation in HEK293FT cells expressing N-terminally (SF-MTDH) or C-terminally (MTDH-SF) tagged MTDH. Data are normalized to SF-MTDH.
(B) Immunofluorescence staining of SF-MTDH and calnexin in HeLa cells. Scale bar, 5 μm.

(C) Predicted structure of the N-terminal 90 amino acids of MTDH from AlphaFold2, highlighting residues 1–5 (green), α-helix (orange), flexible loop (blue), and TMD (yellow).

(D) AlphaFold3-predicted structures of MTDH-ΔHelix (left) and MTDH-ΔLoop (right).

(E) Schematic of MTDH-ADD mutant.

(F) Immunofluorescence staining of MTDH-ADD-SF and calnexin in HeLa cells. Scale bar, 5 μm.

(G) APE assay of *S*-palmitoylation in HEK293FT cells expressing MTDH-WT or MTDH-ADD. Data are normalized to WT (+HAM).

(H, I) AlphaFold3-predicted structures of SF-MTDH (H) and MTDH-ADD (I); SF tag is shown in purple.

Experiments in (A), (B), (F), and (G) were independently repeated at least three times.

**
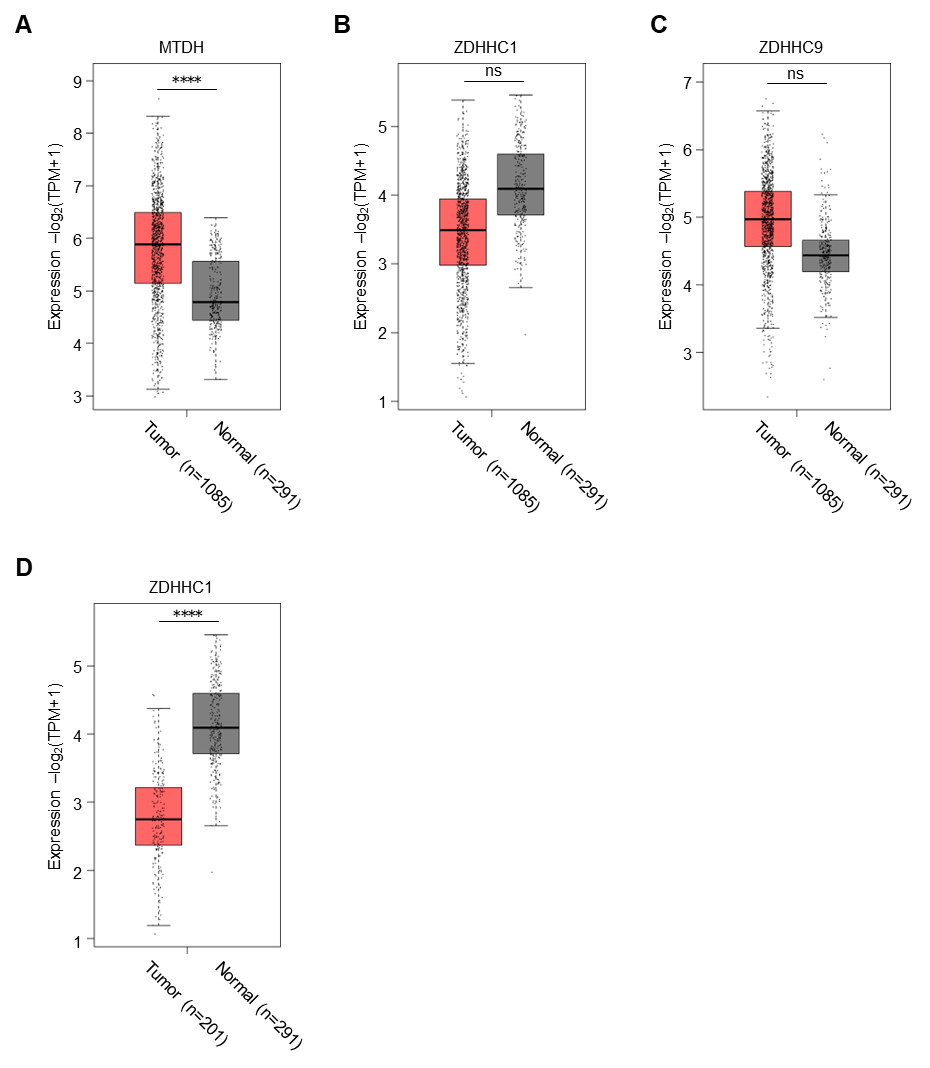
**

**Figure S3. Gene expression analysis of MTDH, ZDHHC1, and ZDHHC9.**

(A–C) Expression levels of MTDH (A), ZDHHC1 (B), and ZDHHC9 (C) analyzed by GEPIA using TCGA BRCA and GTEx datasets.

(D) Expression analysis of ZDHHC1 in estrogen receptor-negative BRCA (TCGA and GTEx) by GEPIA.

Log2FC cut-off = 1; differential analysis by one-way ANOVA; ****p < 0.0001.

**
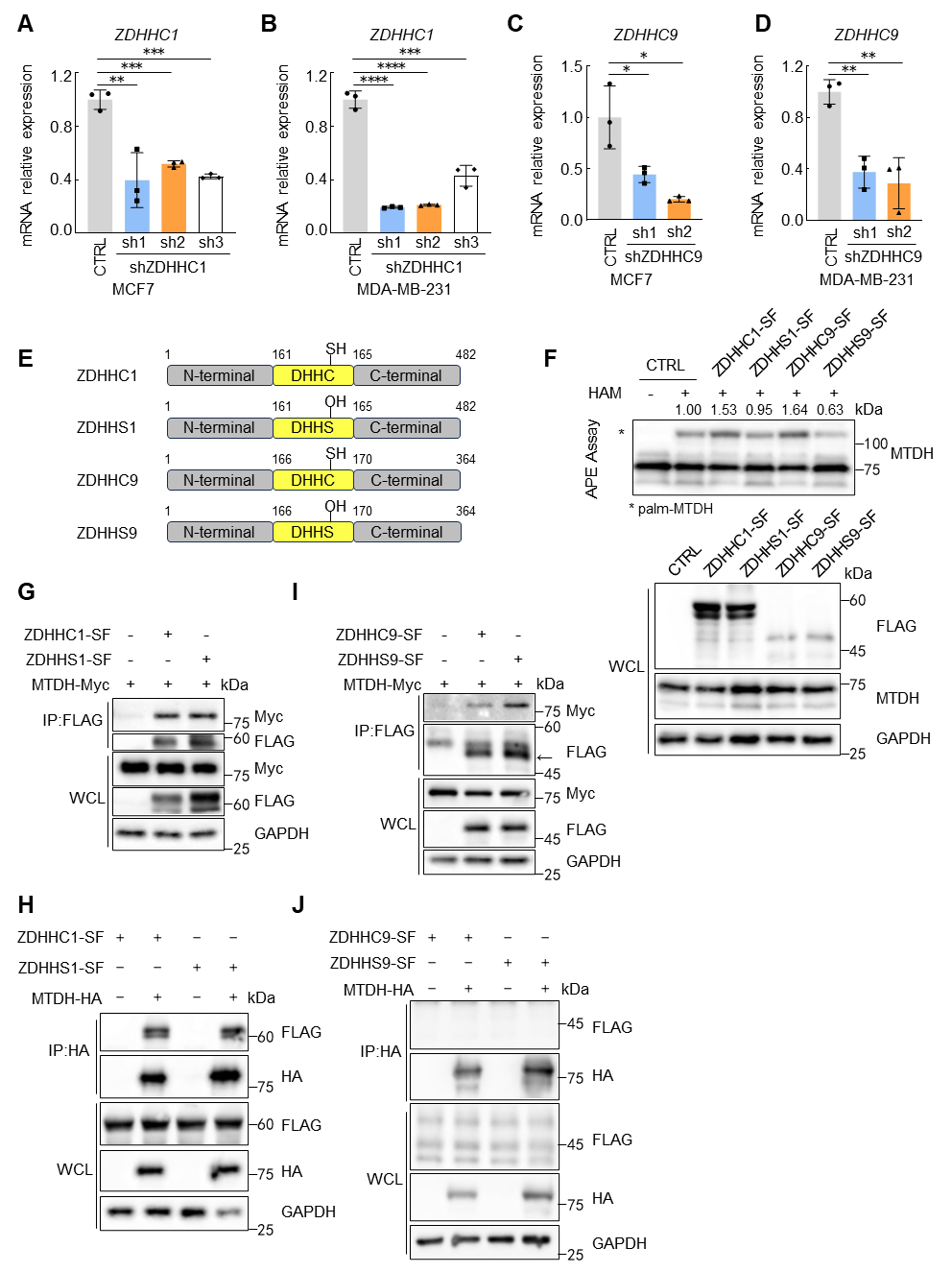
**

**Figure S4. ZDHHC1 and ZDHHC9 are the palmitoyl acyltransferases of MTDH.**

(A–D) qRT-PCR analysis of ZDHHC1 (A, B) and ZDHHC9 (C, D) mRNA in MCF7 and MDA-MB-231 cells transduced with control or shRNAs targeting ZDHHC1/9.

(E) Schematic of catalytically inactive mutants ZDHHS1 and ZDHHS9.

(F) APE assay of endogenous MTDH *S*-palmitoylation in HEK293FT cells expressing SF-ZDHHC1, ZDHHS1, ZDHHC9, or ZDHHS9.

(G, H) Co-immunoprecipitation of MTDH with ZDHHC1-SF or ZDHHS1-SF in cells co-expressing MTDH-Myc (G) or MTDH-HA (H).

(I, J) Co-immunoprecipitation of MTDH with ZDHHC9-SF or ZDHHS9-SF in cells co-expressing MTDH-Myc (I) or MTDH-HA (J).

Data represent biological replicates with s.d. error bars. *p < 0.05, **p < 0.01, *** p < 0.001, **** p < 0.0001 by two-tailed, unpaired Student’s *t*-test. All experiments were repeated at least three times.


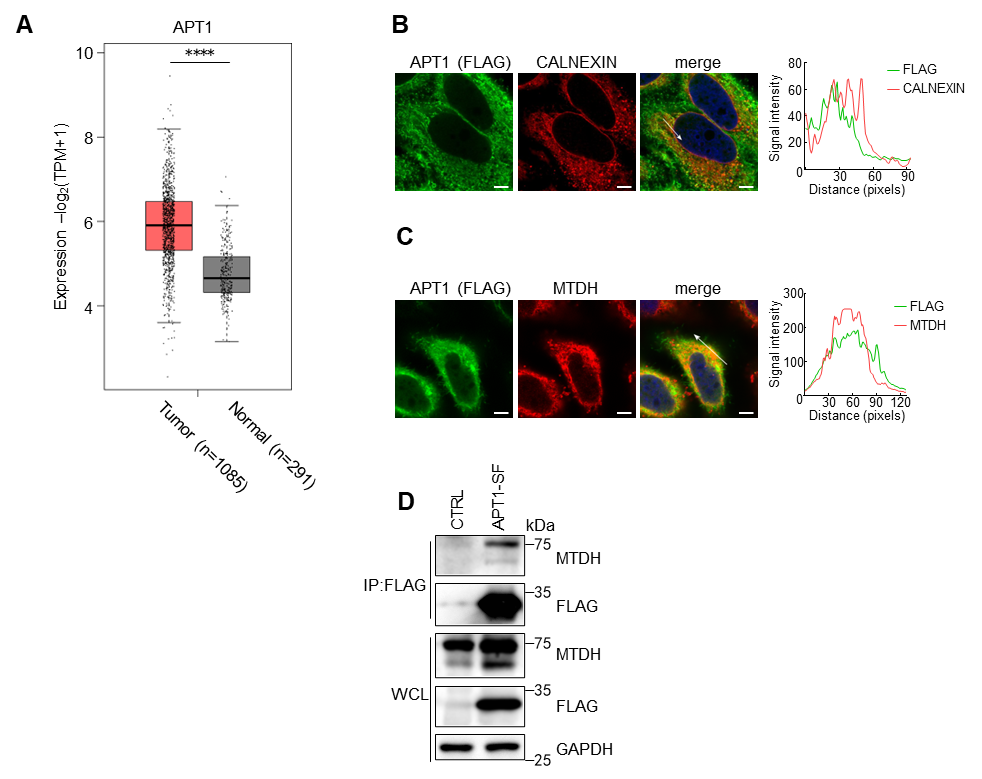


**Figure S5. MTDH is *S*-depalmitoylated by APT1.**

(A) APT1 expression analysis in BRCA from TCGA and GTEx datasets using GEPIA. Log2FC cut-off = 1; differential analysis by one-way ANOVA; **** p < 0.0001.

(B) Immunofluorescence staining of APT1-SF and calnexin in HeLa cells. Scale bar, 5 μm.

(C) Immunofluorescence staining of APT1-SF and MTDH in HeLa cells. Scale bar, 5 μm.

(D) Co-immunoprecipitation of APT1-SF with endogenous MTDH in HEK293FT cells.

Experiments in (B), (C) and (D) were repeated at least three times independently.

**
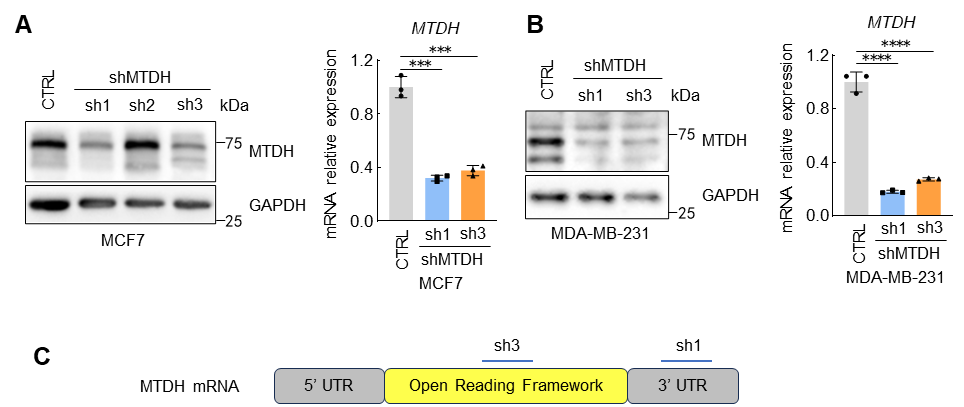
**

**Figure S6. Loss of MTDH *S*-palmitoylation enhances the cell migration ability.**

(A, B) Immunoblot of MTDH protein and qRT-PCR analysis of MTDH mRNA in MCF7 (A) and MDA-MB-231 (B) cells infected with control or MTDH-targeting shRNAs.
(C) Schematic diagram of the MTDH shRNA targeting region.

Data are presented as mean ± s.d. from biological replicates. *** p < 0.001, **** p < 0.0001 by two-tailed, unpaired Student’s *t*-test. All experiments were repeated at least three times.

**
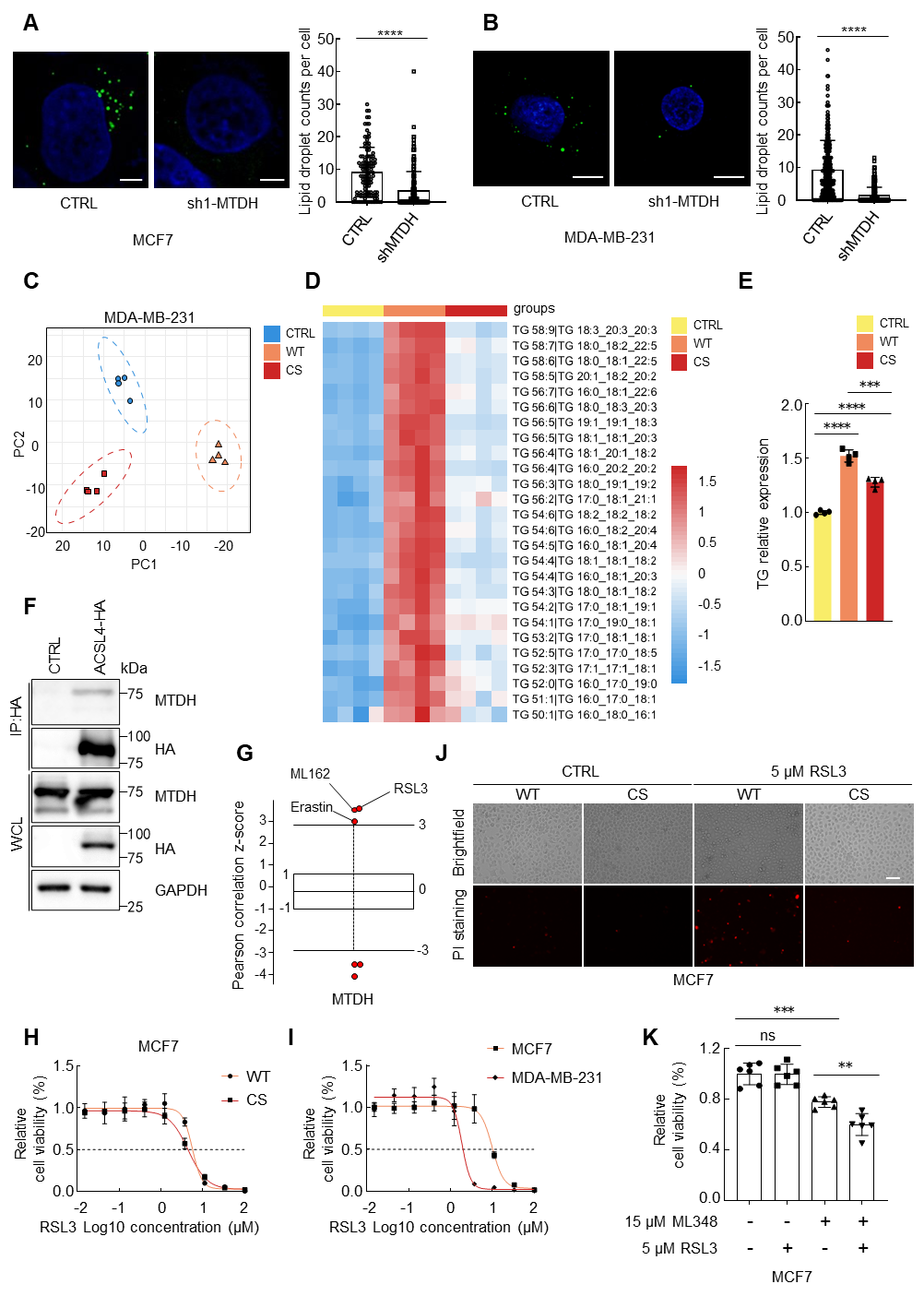
Figure S7. Loss of MTDH *S*-palmitoylation enhances breast cancer cells ferroptosis resistance.**

(A, B) Confocal images of BODIPY 493/503-stained lipid droplets in MCF7 (A) and MDA-MB-231 (B) cells expressing control or shMTDH. Quantification performed using CellProfiler (v4.2.5). Scale bar, 5 μm. **** p < 0.0001, *n* > 100 cells.

(C) PCA of lipidomics profiles in MTDH-knockdown MDA-MB-231 cells expressing empty vector, MTDH-WT, or MTDH-CS. (*n* = 4 biological independent samples).

(D) Heatmap of triglyceride (TG) species in cells from (C); data normalized to species means. (*n* = 4 biological independent samples).

(E) Quantification of total TG species in corresponding cells. Data normalized to CTRL. Two-tailed Student’s *t*-test; ***p < 0.001, ****p < 0.0001. (*n*=4 biological independent samples).

(F) Co-immunoprecipitation of ACSL4-HA with endogenous MTDH in HEK293FT cells.

(G) Pearson correlation analysis of MTDH expression and sensitivity to ferroptosis inducers (RSL3, ML162, Erastin) from CTRP database.

(H) Cell viability assay of MCF7 MTDH-knockdown cells expressing MTDH-WT or MTDH-CS treated with RSL3 for 48 h.

(I) Cell viability of MCF7 and MDA-MB-231 cells treated with RSL3 for 48 h.

(J) Images of MTDH-knockdown MCF7 cells expressing WT or CS, treated with RSL3 for 24 hours in the presence of PI.

(K) Cell viability analysis of MCF7 cells, treated with or without ML348 or RSL3 for 24 hours. Data normalized to the group without ML348 and RSL3. Two-tailed Student’s *t*-test; **p < 0.01, ***p < 0.001. (*n*=3 biological independent samples)

Data in (H, I) are presented as mean ± s.d. from biological replicates. All experiments were independently repeated at least three times. Statistical analysis was performed using a two-tailed, unpaired Student’s *t*-test.
